# Clathrin Facilitates the Morphogenesis of Retrovirus Particles

**DOI:** 10.1371/journal.ppat.1002119

**Published:** 2011-06-30

**Authors:** Fengwen Zhang, Trinity Zang, Sam J. Wilson, Marc C. Johnson, Paul D. Bieniasz

**Affiliations:** 1 Laboratory of Retrovirology, Aaron Diamond AIDS Research Center, The Rockefeller University, New York, New York, United States of America; 2 Howard Hughes Medical Institute, Aaron Diamond AIDS Research Center, The Rockefeller University, New York, New York, United States of America; 3 Department of Molecular Microbiology and Immunology, M616 Medical Sciences Building, University of Missouri-Columbia, Columbia, Missouri, United States of America; University of Geneva, Switzerland

## Abstract

The morphogenesis of retroviral particles is driven by Gag and GagPol proteins that provide the major structural component and enzymatic activities required for particle assembly and maturation. In addition, a number of cellular proteins are found in retrovirus particles; some of these are important for viral replication, but many lack a known functional role. One such protein is clathrin, which is assumed to be passively incorporated into virions due to its abundance at the plasma membrane. We found that clathrin is not only exceptionally abundant in highly purified HIV-1 particles but is recruited with high specificity. In particular, the HIV-1 Pol protein was absolutely required for clathrin incorporation and point mutations in reverse transcriptase or integrase domains of Pol could abolish incorporation. Clathrin was also specifically incorporated into other retrovirus particles, including members of the lentivirus (simian immunodeficiency virus, SIVmac), gammaretrovirus (murine leukemia virus, MLV) and betaretrovirus (Mason-Pfizer monkey virus, M-PMV) genera. However, unlike HIV-1, these other retroviruses recruited clathrin primarily using peptide motifs in their respective Gag proteins that mimicked motifs found in cellular clathrin adaptors. Perturbation of clathrin incorporation into these retroviruses, via mutagenesis of viral proteins, siRNA based clathrin depletion or adaptor protein (AP180) induced clathrin sequestration, had a range of effects on the accuracy of particle morphogenesis. These effects varied according to which retrovirus was examined, and included Gag and/or Pol protein destabilization, inhibition of particle assembly and reduction in virion infectivity. For each retrovirus examined, clathrin incorporation appeared to be important for optimal replication. These data indicate that a number of retroviruses employ clathrin to facilitate the accurate morphogenesis of infectious particles. We propose a model in which clathrin contributes to the spatial organization of Gag and Pol proteins, and thereby regulates proteolytic processing of virion components during particle assembly.

## Author Summary

The assembly and maturation of infectious retroviruses is driven by two viral proteins, Gag and Pol. Additionally, a number of cellular proteins are found in retrovirus particles, many of which lack a known functional role. One such protein is clathrin, which normally mediates several physiological processes in cells and was previously thought to be only passively incorporated into virions. In this study we show that clathrin is actively, specifically and abundantly incorporated into retrovirus particles. In several cases, retroviral proteins encode peptide motifs that mimic those found in cellular adaptor proteins that are responsible for clathrin recruitment. The range of retroviruses into which clathrin is packaged includes human and simian immunodeficiency viruses as well as other murine and simian retroviruses. Manipulations that prevented clathrin incorporation into virions also caused a variety of defects in the genesis of infectious retroviruses, including viral protein destabilization, inhibition of particle assembly and release, and reduction in virion infectiousness. The precise nature of the defect varied according to which particular retrovirus was examined. Overall these studies suggest that clathrin is frequently employed by retroviruses to facilitate the accurate assembly of infectious virions.

## Introduction

To establish a productive infection in host cells, retroviruses have evolved strategies that employ numerous host factors to facilitate their replication. Recently, several groups have applied genome-wide RNAi screens to identify hundreds of candidate host factors that may facilitate human immunodeficiency virus-1 (HIV-1) and murine leukemia virus (MLV) infection [1], [2], [3]. Other strategies to identify host factors that facilitate virus replication include the identification of proteins that bind to viral proteins [4] and analysis of the proteomes that are incorporated into virions [5], [6], [7]. Indeed, host proteins involved in HIV-1 budding, such as Tsg101 [8] and ALIX [9] can be found in virions. Additionally, proteins that modulate virion infectivity such as cyclophilin A (CypA) [10], and Hsc70 [11] can also be demonstrated to be virion components. However, while proteomic analyses of purified HIV-1 or MLV particles have revealed dozens of virion-associated host proteins, no biological significance has been attached to the virion association of many of them. One such protein is clathrin, which previous reports suggest is only passively incorporated into particles [6].

Clathrin has been intensively studied in the context of cell biology (reviewed in [12]). It is a cytosolic protein that functions in vesicle genesis and transport and, specifically, mediates endocytosis from the plasma membrane and cargo trafficking from the *trans*-Golgi network (TGN). Clathrin is comprised of a trimer of 180 kDa heavy chains (HC) that are arranged with their N-terminal adaptor binding domains at the extremities of each leg of a triskelion, while clathrin light chains (LC) bind to heavy chains close to their C-termini. Clathrin adaptors (such as the AP family) govern the sorting of specific cargoes into clathrin-coated vesicles and recruitment of clathrin to membranes (reviewed in [13]). Many of these adaptors contain motifs such as LΦXΦ[DE] (Φ indicates a bulky hydrophobic residue), or in the case of AP180, repeated motifs with the sequence DLL, which bind to the clathrin N-terminal β–propeller domain, and facilitate the recruitment of clathrin to the plasma membrane [14], [15].

Here, we demonstrate that clathrin is abundantly and specifically incorporated into a range of diverse retrovirus particles, including HIV-1, SIVmac, MLV and M-PMV through interactions with Gag or Pol proteins. Indeed, several retroviral Gag proteins were found to encode peptide motifs that drive clathrin incorporation and mimic those found in cellular clathrin adaptor proteins. We also show that mutations in the motifs that mediate clathrin recruitment have a range of effects on the accuracy of particle morphogenesis, or on virion infectiousness, depending on the particular retrovirus that was examined. In several cases, we demonstrate that these effects can be recapitulated by reducing the available levels of clathrin in cells.

## Results

### Clathrin is abundantly and specifically incorporated into HIV-1 particles

**Figure 1:**
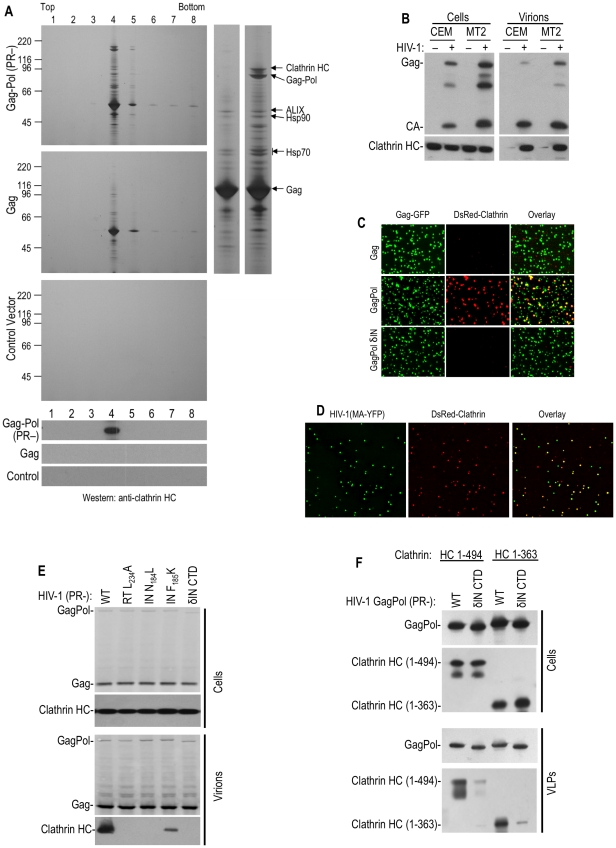
Clathrin is recruited into HIV-1 particles by Pol. (A) Particles from HIV-1 GagPol or Gag expressing, or mock transfected, cells were pelleted through 20% sucrose, treated with subtilisin and separated on Optiprep gradients. Particulate material in gradient fractions was analyzed by silver staining (upper three panels). An expanded view of lane 4 from the gradients containing Gag and GagPol particles is shown to the right, with salient protein identifications by mass spectroscopy. The lower three panels show a Western blot analysis, using an anti-clathrin HC antibody. (B) Western blot analysis of CEM and MT2 cells and corresponding VLPs generated following single cycle infection (40 hrs) with Env-deleted, VSV G-pseudotyped HIV-1, using anti-HIV-1 CA (upper panels) and anti-clathrin HC antibodies (lower panels). Note that the virion loading represent 1/5 of the total sample, while the cell lysate represents 1/100 of the total sample, and exposure times were the same for cell lysates and virions. (C) 293T cells stably expressing DsRed-clathrin LC were transfected with plasmids expressing HIV-1 Gag, protease-defective GagPol (D25A), or IN-deleted GagPol, spiked in a 1∶4 ratio with a plasmid expressing Gag-GFP. Particles were recovered through 20% sucrose, filtered, placed on a poly-Lysine coated coverslip and imaged. The fields shown are 5 µm square. (D) Similar to C, except that the 293T cells stably expressing DsRed-clathrin LC were transfected with an HIV-1 (MA-YFP) proviral plasmid and images are 10 µm squares. (E) Requirements for clathrin HC packaging into HIV-1 particles. 293T cells were transfected with various protease-defective (D25A) HIV-1 proviral plasmids, that were otherwise either wild-type, or bore additional mutations in RT (L234A), IN (N184L, F185K) or an IN δCTD deletion. Cells and virions were analyzed by Western blotting with anti-HIV-1 CA and clathrin HC antibodies. (F) Incorporation of N-terminal β-propeller domain of clathrin HC (1–494aa or 1–363aa) into HIV-1 GagPol-derived VLPs. Plasmids encoding HA-tagged clathrin HC N-terminal domain (1–494aa or 1–363aa) were co-transfected with wild-type or IN δCTD HIV-1 protease-inactive GagPol expression plasmids. Cells and VLPs were analyzed by Western blotting with anti-HIV-1 CA and anti-HA antibodies.

Initially, to discover potential host factors involved in HIV-1 replication, we set out to identify cellular proteins that are incorporated into HIV-1 particles, using a slightly different strategy compared to previous studies. To minimize the “noise” in such experiments, such as contaminating cell debris, or passively incorporated proteins, and prevent degradation of incorporated cellular proteins by the viral protease, plasmids expressing codon-optimized HIV-1 Gag alone, or protease-inactive (D25A, PR-) GagPol proteins were transfected into 293T cells. This generated a very high yield of virus-like particles (VLPs) without apparent cytotoxicity. Silver and Coomassie blue staining of SDS-PAGE gels (Figure 1A), loaded with iodixinal gradient-purified GagPol VLPs revealed that five cellular proteins were abundantly incorporated into GagPol VLPs and were resistant to digestion by externally applied subtilisin. Mass spectroscopic analysis of the excised bands identified these proteins as CypA, Hsp70, Hsp90, AIP1/ALIX and clathrin HC (Figure 1A), all of which have previously been identified as components of HIV-1 particles [5], [6], [9], [10], [11]. Strikingly, and in contrast to the other proteins that were found in HIV-1 GagPol VLPs, both silver staining and Western blot analysis revealed that clathrin HC was undetectable in particles simultaneously generated using the HIV-1 Gag protein alone (Figure 1A). Moreover, visual inspection of silver or Coomassie stained gels suggested that clathrin HC was packaged into GagPol VLPs at exceptionally high level, i.e. the level of clathrin HC in virions approached that of GagPol (Figure 1A, right panel). To verify this finding with authentic virion particles, the T-cell lines CEMX174 and MT2 were infected with VSV-G pseudotyped, Env-defective HIV-1 at a multiplicity of infection of ∼1, washed extensively and progeny virions were harvested 40 h later (Figure 1B). Western blot analysis revealed that clathrin HC was abundantly incorporated into these virions. Indeed, in experiments where virions were simultaneously generated via infection of T-cell lines or transfection of 293T cells and the amount of clathrin incorporated into virions directly compared by quantitative fluorescence-based Western blotting (LI-COR), we found that the T-cell derived virions incorporate as much or more clathrin than the 293T-derived particles (Figure S1). Moreover, we determined that 6 to 8% of the total clathrin HC in the HIV-1 infected T-cell cultures appeared to be present in extracellular virions rather than cells (Figure S1).

Consistent with previous findings that clathrin LC binds to the C-terminal portion of clathrin HC and forms a stable complex with it [12], clathrin LC was also incorporated into HIV-1 virions. Indeed, when green fluorescent HIV-1 VLPs were generated by coexpression of HIV-1 GagGFP and GagPol in cells stably expressing fluorescently tagged clathrin LC (DsRed-clathrin LC), about 40% of the VLPs were labeled with sufficient DsRed-clathrin LC to be visualized by deconvolution microscopy (Figure 1C, Table S1). In contrast, only a few VLPs generated by coexpression of GagGFP and Gag were red-fluorescent. In similar experiments, authentic virions were generated by transfection with a proviral plasmid encoding YFP embedded in the stalk region of the MA domain of Gag into cells stably expressing DsRed-clathrin LC. In this case, 70–80% of YFP+ virions contained sufficient DsRed-clathrin LC to be visualized as colocalizing red fluorescent puncta (Figure 1D, Table S1).

### HIV-1 Pol is responsible for clathrin incorporation into virions

Given that clathrin incorporation into HIV-1 particles appeared Pol-dependent, we next attempted to map the sequences responsible for its incorporation, using particles generated by expressing protease-defective GagPol proteins and detection of virion associated clathrin using Western blot or microscopy assays. Precise deletion of the reverse transcriptase (RT) or integrase (IN) domain in this context did not markedly affect VLP release but, surprisingly, both manipulations abolished clathrin incorporation (Figure 1C and Table S1). Similarly, several Pol truncations or point mutations, constructed in the context of protease-defective proviral plasmid, also inhibited or abolished clathrin incorporation (Figure 1E and Table S1). Specifically, mutations in reverse transcriptase (RT), including L234A, that inhibit RT dimerization [16] prevented clathrin incorporation as did deletion of the IN C-terminal domain (CTD) as well as two so-called ‘class II’ IN mutations, N184L and F185K [17]. Western blot analysis of virions indicated that, while the levels of GagPol protein in cells and virions were not apparently affected by these mutations, clathrin incorporation was inhibited (Figure 1E). We even found that the presence of the non-nucleoside RT inhibitor efavirenz, which stimulates RT dimerization [16], during the production of VLPs, inhibited clathrin incorporation into virions (Table S1). In contrast, no effect on clathrin incorporation was observed for the active site IN point mutant E152K (Figure S2). Taken together, these findings demonstrate that multiple Pol domains are strictly required for clathrin incorporation into HIV-1 VLPs. This suggests that the overall conformation of Pol is critical for clathrin packaging, and effectively made it impractical to map small motifs in Pol that might be responsible for clathrin recruitment.

### The adaptor-binding beta propeller domain of clathrin is packaged into HIV-1 particles

Clathrin adaptors GGA and AP-1, 2 and 3 are known to bind to the N-terminal 7-bladed β–propeller domain of clathrin HC [13]. This clathrin domain, encoded by residues 1–494aa is known to fold autonomously when expressed in the absence of other clathrin domains [18]. Co-expression of N-terminal 1–494aa or 1–363aa fragments of clathrin along with HIV-1 GagPol resulted in specific incorporation of these clathin fragments into wild-type VLPs, but not into IN δCTD VLPs (Figure 1F), mimicking the property of endogenous full-length clathrin. Thus, the N-terminal adaptor binding domain of clathrin HC was sufficient to drive its incorporation into HIV-1 GagPol VLPs.

### Clathrin incorporation into SIVmac particles is driven primarily by Gag, with a minor contribution from Pol

**Figure 2:**
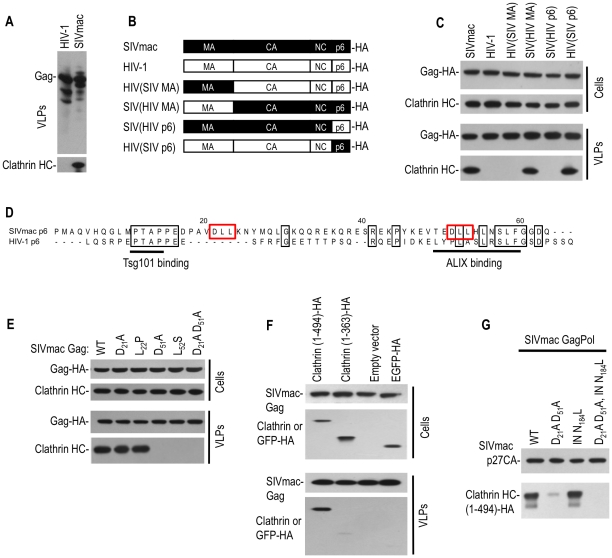
Clathrin is recruited into SIVmac particles by Gag (p6) with a minor contribution from Pol. (A) Western blot analysis of VLPs generated using HIV-1 and SIVmac Gag expression plasmids. Blots were probed with anti-HIV-1 CA (upper) or anti-clathrin HC (lower) antibodies. (B, C) The p6 domain of SIVmac gag mediates clathrin incorporation. A panel of chimeras were constructed using HIV-1 Gag and SIVmac Gag proteins and encoded an HA epitope at their C-termini (B). Cell lysates and VLPs, harvested after transfection of the constructs depicted in (B) were analyzed by Western blotting (C) with anti-HA and anti-clathrin HC antibodies. (D) Alignment of HIV-1(NL4-3) and SIVmac239 p6 sequences. Identities are boxed in black, and putative binding motifs are highlighted by underlining or red boxes. (E) DLL motifs in p6 recruit clathrin into SIVmac Gag VLPs. 293T cells were transfected with wild-type or the indicated mutant SIVmac Gag expression plasmids. Cells and VLPs lysates were probed with anti-Gag and anti-clathrin HC antibodies. (F) Incorporation of the N-terminal adaptor binding domain of clathrin HC into SIVmac Gag VLPs. Plasmids encoding an HA-tagged 1–494aa, or 1–363aa clathrin N-terminal domain or EGFP-HA were co-transfected with an SIVmac Gag expression plasmid. Cells lysates and VLPs were probed with anti-CA and anti-HA antibodies. (G) Incorporation of clathrin HC into virions via SIVmac Gag and Pol. A plasmid expressing an HA tagged N-terminal clathrin HC domain (1–494aa) was co-transfected with SIVmac GagPol expression plasmids encoding wild-type, DLL mutant, integrase mutant (N184L) or both mutations, respectively. Virion lysates were probed with anti-CA and anti-HA antibodies.

To determine whether clathrin incorporation into virions is a general feature of primate lentiviruses, we next determined whether it also occurred in SIVmac. In contrast to results obtained with HIV-1 Gag, analysis of VLPs generated using only SIVmac Gag showed that clathrin was abundantly incorporated into VLPs in the absence of Pol (Figure 2A). Mapping experiments in which chimeric SIVmac/HIV-1 Gag proteins (Figure 2B) were used to generate VLPs revealed that Gag proteins encoding the SIVmac p6 domain, specifically SIV(HIV MA) and HIV(SIV p6), yielded VLPs containing clathrin (Figure 2B and 2C lane 4 and lane 6). Conversely, the reciprocal chimeric Gag proteins, HIV(SIV MA) and SIV(HIV p6), generated VLPs that did not incorporate clathrin (Figure 2B and 2C lane 3 and lane 5). Thus, the differential abilities of HIV-1 and SIVmac Gag VLPs to incorporate clathrin were clearly governed by the p6 domain.

Inspection of the SIVmac p6 protein sequence revealed two ‘DLL’ motifs (positioned at p6 residues 21–23 and 51–53) that are absent in HIV-1 p6 (Figure 2D). Because the clathrin adaptor AP180 employs multiple copies of a DLL motif in its C-terminal domain to directly bind to clathrin HC [15], we mutated either or both DLL motifs in SIVmac p6 and tested the ability of the mutant Gag proteins to drive clathrin incorporation into VLPs. Western blot analysis indicated that mutations in both motifs (D21A, D51A) or the second motif only (D51A or L52S) reduced clathrin incorporation to almost undetectable levels, while mutation in the first DLL motif alone had little effect (Figure 2E).

To determine the clathrin domain that mediates packaging into SIVmac Gag VLPs, expression plasmids encoding the 1–494aa or 1–363aa clathrin HC N-terminal domains were coexpressed with SIVmac Gag. As shown in Figure 2F, SIVmac VLPs efficiently incorporated the clathrin HC N-terminal 1–494aa fragment, but unlike HIV-1, the 1–363aa fragment was poorly incorporated (Figure 2F, bottom panel). The significance of the difference in clathrin sequence requirements for incorporation into HIV-1 versus SIVmac virions is unclear at present, but in both cases the N-terminal adaptor binding domain appeared to be responsible for driving incorporation.

Next, we tested whether SIVmac Pol, like HIV-1 Pol, could also drive clathrin incorporation. To facilitate clathrin detection, an HA tagged clathrin N-terminal domain (1–494aa) was co-transfected with plasmids expressing either wild-type SIVmac GagPol, or mutants in which either (i) the DLL motifs in p6 were mutated (DLL- GagPol), (ii) IN was mutated (N184L) in a way analogous to that which blocks clathrin incorporation into HIV-1 GagPol VLPs, or (iii) both p6 and IN were mutated. SIVmac DLL- GagPol exhibited greatly diminished clathrin packaging into VLPs (Figure 2G). However, some clathrin incorporation was observed in SIVmac DLL- GagPol VLPs, and this incorporation was completely abolished by additional mutation at IN residue N184 (Figure 2G). Thus, in SIVmac, both Gag and Pol contribute to clathrin incorporation into virions, but Gag appears to play the dominant role and drives significantly more clathrin incorporation than Pol.

### Clathrin incorporation into gammaretrovirus and betaretrovirus particles

**Figure 3:**
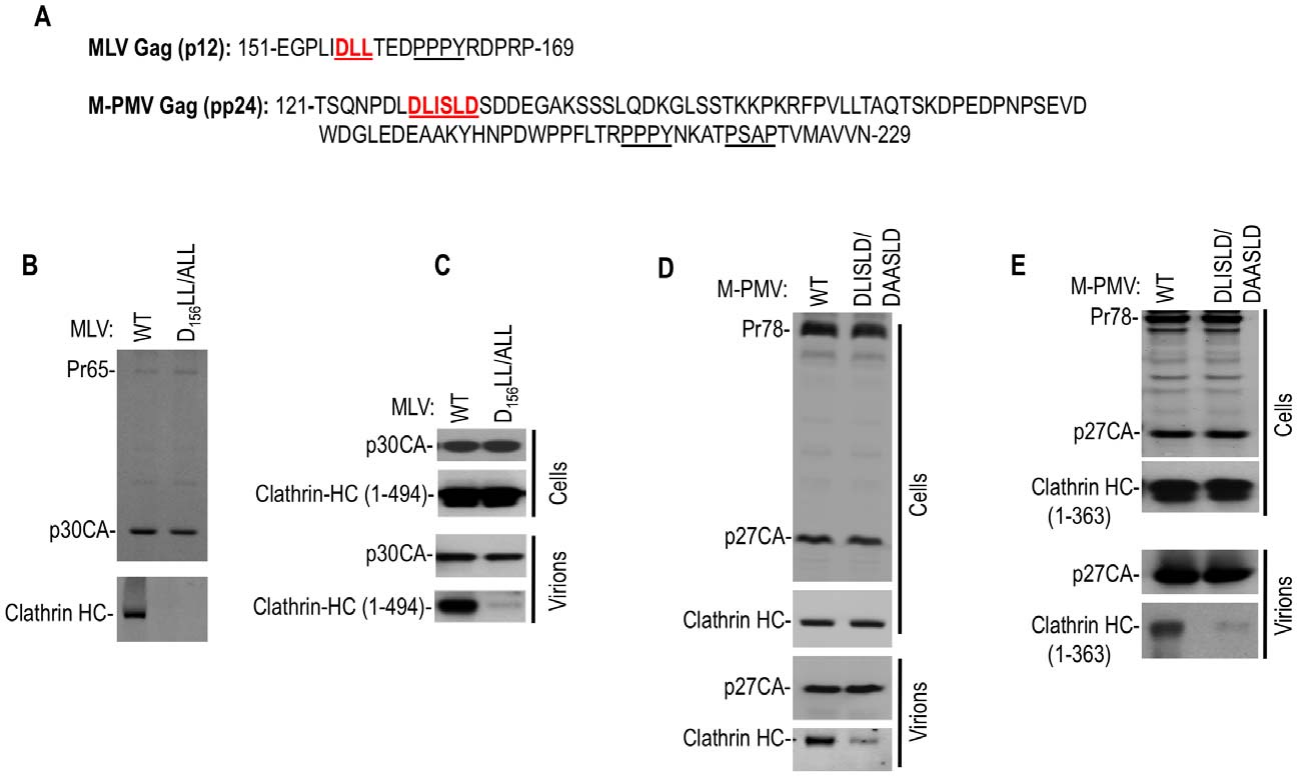
Clathrin is incorporated into MLV and M-PMV virions. (A) Putative clathrin binding sites proximal to L-domain sequences in MLV p12 and M-PMV pp24 Gag proteins. (B and C) MLV particles incorporate clathrin HC or a coexpressed N-terminal 1–494aa clathrin HC fragment via D_156_L_157_L_158_ motif in the p12 domain. Wild-type or DLL/ALL mutant MLV GagPol expression plasmids were transfected alone (B) or with a plasmid encoding HA tagged clathrin HC 1–494aa (C) in 293T cells. Pelleted virions (B) or cell lysates and virions (C) were subjected to Western blot analysis with anti-MLV CA and anti-clathrin HC (B) or anti-HA antibodies (C). (D and E) M-PMV particles incorporate endogenous clathrin HC or a N-terminal 1–363aa clathrin HC fragment via a D_128_LISLD_133_ motif in the pp24 domain. Wild-type or DLISLD/DAASLD mutant M-PMV proviral plasmids (pSARM-4) were transfected alone (D) or with a plasmid encoding an HA tagged clathrin HC 1–363aa (E). Cell lysates and virions were subjected to Western blot analysis with anti-M-PMV CA and anti-clathrin HC (D) or anti-HA (E) antibodies.

The finding that clathrin was specifically packaged into HIV-1 and SIVmac VLPs prompted us to ask whether clathrin incorporation is a general property of retroviruses. Inspection of a variety of retroviral Gag protein sequences revealed that some, but not all, encoded putative clathrin binding peptides, including DLL and LLTLD motifs in their Gag proteins. In particular, a prototype gammaretrovirus, MLV and a prototype betaretrovirus, M-PMV were selected for further investigation. Putative clathrin binding motifs, DLL and DLISLD respectively, were found in their Gag proteins proximal to their late domains (a DLL motif at 156–158aa in MLV Gag and a DLISLD motif at 129–133aa of M-PMV Gag respectively, Figure 3A). Each of these viruses was found to package either endogenously expressed clathrin HC, or coexpressed N-terminal adaptor-binding domain fragments of clathrin HC (1–494aa or 1–363aa, Figure 3B,C,D,E) into virions. This incorporation was specific because mutations in respective putative clathrin binding sites in Gag (DLL to ALL in MLV or DLISLD to DAASLD in M-PMV) dramatically reduced this incorporation. Notably, the yield of virion particles was unaffected by mutations in these clathrin recruiting motifs (Figure 3B,C,D,E). Mutations in other candidate clathrin HC binding sites in Gag (_533_LLTLD_537_ at the C-terminus of MLV Gag and _30_DLL_32_ in the matrix domain of M-PMV, respectively) caused no reduction in clathrin incorporation, indicating that these other candidate motifs do not play a critical role in clathrin packaging (unpublished observations).

Overall, these findings demonstrated that clathrin can be specifically incorporated into virions from widely divergent retroviruses, and that this incorporation is driven by the adaptor-binding domain at the N-terminus of clathrin HC. Moreover, clathrin incorporation sometimes occurs via the action of peptide motifs in viral structural proteins that mimic those found in cellular clathrin adaptors.

### Involvement of clathrin in the genesis of infectious retrovirus particles

To probe the role of clathrin in retrovirus replication, we adopted a variety of approaches, including the analysis of viral mutants that were defective for clathrin incorporation, as well as depletion of clathrin using siRNA based approaches. Attempts to deplete clathrin using siRNA were complicated by the fact that it is highly abundant, has a relatively long half-life (20 h–50 h) [19], [20] and is essential for various cellular functions and for cell viability. Therefore, while we were able to reproducibly deplete about 70–80% of endogenous clathrin HC (Figure S3A), it proved nearly impossible to completely deplete the intracellular clathrin to a sufficient extent such that a clathrin-deficient viral phenotype could be analyzed. Moreover, since clathrin plays a critical role in a number of cellular pathways, including trafficking of proteins through the secretory pathway, distinguishing the direct effects of depletion from indirect effects is challenging. Therefore, in order to investigate the role that clathrin plays in retrovirus life cycles, we employed multiple approaches including characterizing viruses with mutations that prevent clathrin incorporation, reducing clathrin expression by siRNA depletion (Figure S3A), and overexpressing the C-terminal domain of the clathrin adaptor AP180 to induce clathrin sequestration (Figure S3B) [21], [22].

### Clathrin incorporation and effects on HIV-1 Pol proteins

**Figure 4:**
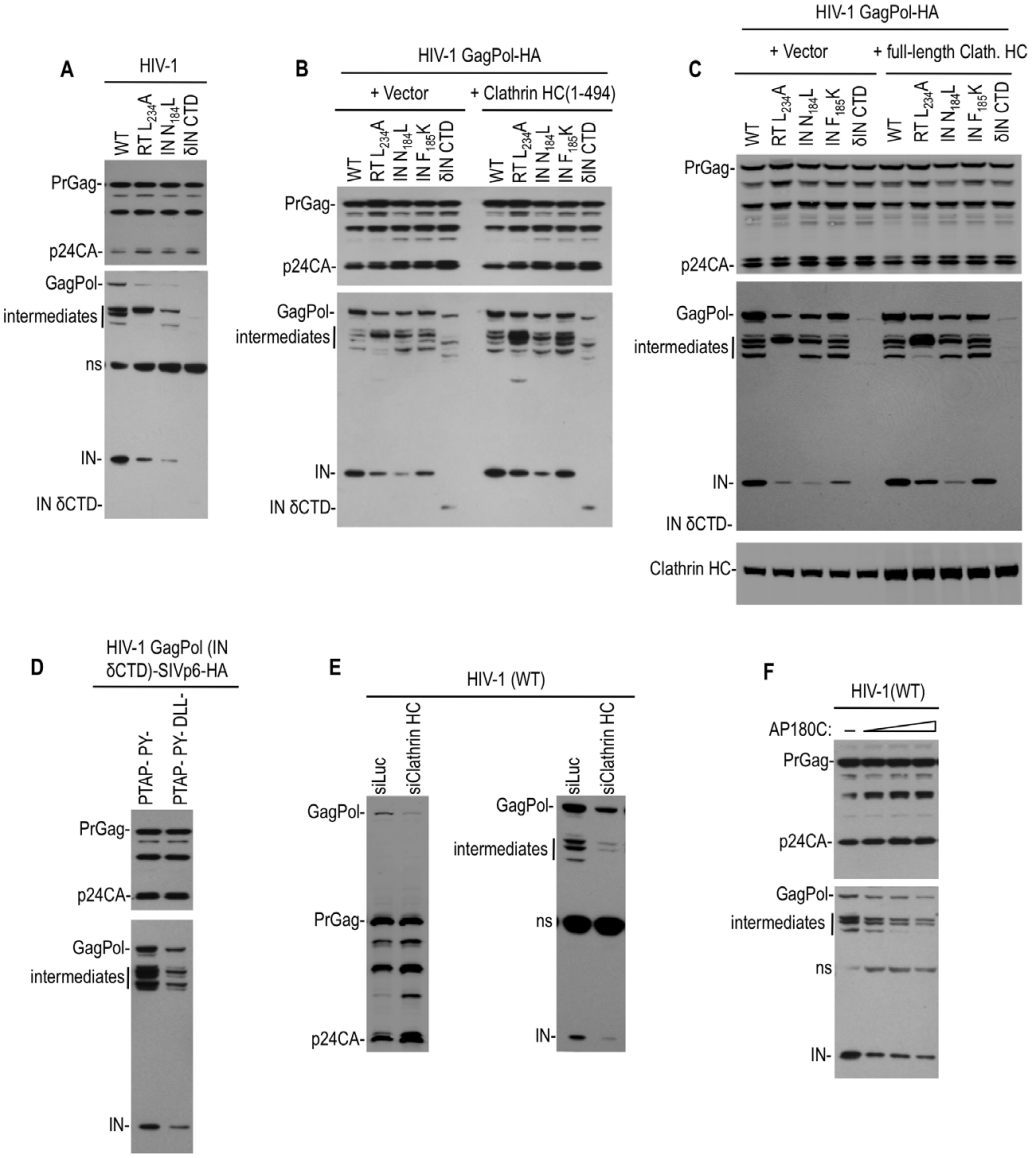
Effect of Pol mutations and clathrin on HIV-1 Pol expression. (A) Apparent instability of Pol protein in the presence of the viral protease caused by mutations that block clathrin recruitment. 293T cells were transfected with wild-type and mutant proviral HIV-1 plasmids. Two days post transfection, cell lysates were analyzed by Western blotting using anti-CA (upper panel) and anti-IN (lower panel) antibodies. “ns” indicates a nonspecific band. (B and C) Increase of Pol protein levels expressed by mutant HIV GagPol expression plasmid by overexpression of the adaptor binding N-terminal clathrin HC domain (B) or full length clathrin HC (C) *in trans*. 293T cells were transfected WT or mutant HIV-1 GagPol expression plasmids bearing an HA tag at the IN C-terminus, along with an empty vector or plasmids expressing clathrin HC 1–494aa (B) or full length clathrin HC (C). Two days post transfection, cell lysates were probed with anti-CA, anti-HA and anti-clathrin HC antibodies. (D) Increase in Pol protein expression by fusion of a clathrin recruiting motif to the C-terminus of GagPol (IN δCTD). 293T cells were transfected an HIV-1 GagPol IN δCTD expression plasmid bearing an HA epitope tag, and Tsg101/ALIX binding-defective SIVmac p6 domain that did (left lane) or did not (right lane) have intact DLL motifs, at the IN C-terminus. Two days post transfection, cell lysates were probed with anti-CA and anti-HA antibodies. (E) Clathin depletion decreases HIV-1 Pol protein levels. 293T cells were cotransfected with an HIV-1 proviral plasmid and luciferase (siLuc) or clathrin HC (siClathrin HC) targeting siRNAs. Cell lysates were probed with anti-CA (left panel) and anti-IN (right panel) antibodies. (F) Clathrin sequestration decreases HIV-1 Pol protein levels. 293T cells were cotransfected with 200 ng of HIV-1 proviral plasmid and either empty vector or increasing amounts of an AP180C expression plasmid. Cell lysates were probed with anti-CA and anti-IN antibodies.

Several mutations in HIV-1 Pol were found to block clathrin incorporation, including mutations in RT at the dimer interface (L234A), as well as class II and CTD-truncating mutations in IN (N184L and δCTD) (Figure 1E). Unfortunately, these mutations are pleiotropic, and may therefore have multiple effects on viral replication by perturbing the tertiary structure and function of the Pol protein. Although these mutations did not have discernable effects on Gag and GagPol precursor protein levels in cells or VLPs when the viral protease was inactivated by mutation (Figure 1E), these mutations induced aberrant proteolytic cleavage of Pol proteins and reduced levels of GagPol precursor in cells and virions when protease was active [23], [24] (Figure 4A, Figure S4). Specifically, when HIV-1 proviruses were expressed in 293T cells, the WT and Pol-mutant viruses generated similar levels of Gag protein and its processed derivatives, but the levels of GagPol precursor, partly processed intermediates and mature IN proteins, detected using an anti-IN antibody, were diminished in the mutants that failed to package clathrin into virions (Figure 4A, Figure S4). Similarly, in a series of constructs that were made to express GagPol protein with an HA-epitope fused at C-terminus of Pol, reduced GagPol levels were detected using an anti-HA antibody when mutations that blocked clathrin were introduced, and aberrant processed derivatives were detected (Figure 4B).

While the RT and IN mutations both blocked clathrin incorporation and induced PR-dependent Pol depletion, it was not clear whether or how these two effects were causally related to each other, given the pleiotropic nature of these mutations. However, these results did suggest the possibility that clathrin recruitment by Pol might inhibit its viral protease-dependent depletion. To investigate this possibility, we examined Pol processing in cells where the clathrin HC N-terminal domain (1–494aa) or the full-length clathrin HC was overexpressed. Notably, for two Pol mutants, namely RT(L234A) and IN(F185K), clathrin HC (1–494aa) or full length clathrin HC overexpresssion led to an increase in the levels of GagPol derived proteins, including mature IN (Figure 4B and 4C). Conversely clathrin overexpression had no, or only a slight effect, on IN (N184L or δCTD) mutants (Figure 4B and 4C). Importantly, the effects of clathrin HC overexpression were specific to Pol; there was no significant effect on Gag precursor or processed derivative levels. To further explore the idea that clathrin recruitment might stabilize Pol in the presence of an active viral protease, we fused the clathrin recruiting domain from SIVmac Gag, namely p6 (mutated in such a way so as not bind to Tsg101 and ALIX), to the C-terminus of HIV-1 GagPol (IN δCTD). This chimera expressed higher levels of Pol protein expression than an equivalent construct containing the SIVmac p6 domain in which the DLL motifs were mutated (Figure 4D). Together, these results are consistent with the notion that clathrin recruitment results in the stabilization of Pol proteins in the presence of an active viral protease. In support of this idea, Western blot analyses revealed that clathrin HC depletion using siRNA reduced the levels of GagPol precursor, IN, and partly processed intermediate Pol proteins in cells transfected with a WT HIV-1 proviral construct (Figure 4E). Importantly, the levels of Gag protein (which are translated from the same viral mRNA species) were not reduced by this manipulation. Furthermore, AP180C overexpression also reduced the levels of intracellular GagPol, IN and intermediate proteins without affecting Gag levels (Figure 4F), again suggesting that clathrin specifically stabilizes Pol proteins in HIV-1 infected cells.

**Figure 5:**
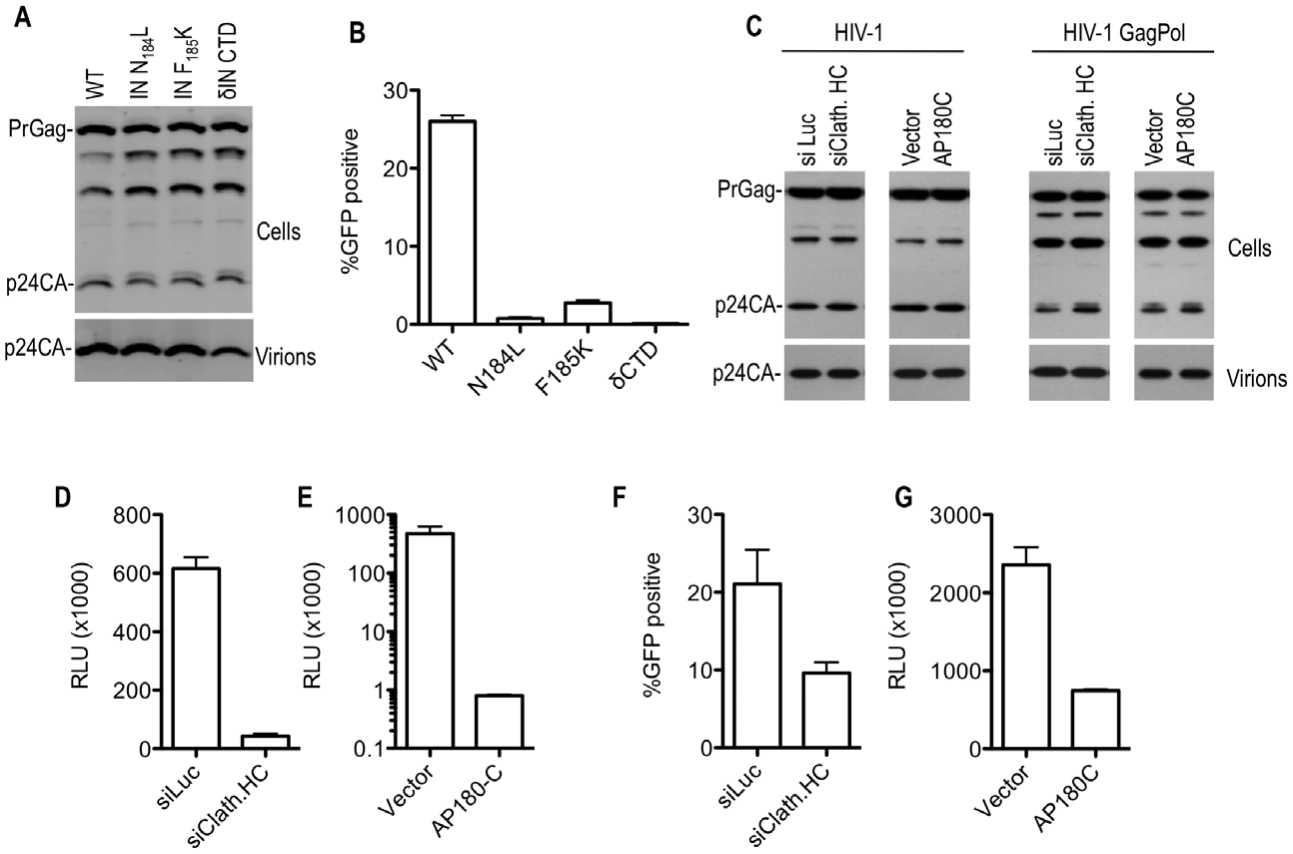
Effect of Pol mutations and clathrin on HIV-1 infectivity. (A) Particle production by 293T cells transfected with WT or IN mutant HIV-1 GagPol expression plasmids. Cell lysates and virions were subjected to Western blot analysis with an anti-CA antibody. (B) Infectious virion production from 293T cells transfected with plasmids expressing WT or mutant HIV-1 GagPol expression plasmids, a GFP-expressing HIV-1 vector and VSV-G. Infectious virus yield was measured using TE671 cells as targets, and FACS analysis to enumerate infected cells. (C) Particle production by 293T cells transfected with an HIV-1 proviral plasmid (left panels) or plasmids expressing WT HIV-1 GagPol, a GFP-expressing HIV-1 vector and VSV-G (right panels). Cells were also transfected with luciferase (siLuc) or clathrin HC (siClath. HC) targeting siRNAs where indicated. Alternatively, cells were cotransfected with an empty plasmid vector or a plasmid expressing AP180C, where indicated. Cell lysates and virions were subjected to Western blot analysis with an anti-CA antibody. (D) Infectious HIV-1 virion production, as measured by TZM-bl indicator assay, after transfection of 293T cells with an intact HIV-1 proviral plasmid (NL4-3) and luciferase or clathrin HC targeting siRNAs. Data are expressed as relative light units (RLU). (E) Infectious HIV-1 virion production, as measured by TZM-bl indicator assay, after transfection of 293T cells with an intact HIV-1 proviral plasmid (NL 4-3) and either empty vector or a plasmid expressing AP180C. Data are expressed as in (D). (F) Infectious virion production from 293T cells transfected with WT HIV-1 GagPol expression plasmids, a GFP-expressing HIV-1 vector and VSV-G. Cells were also transfected with luciferase (siLuc) or clathrin HC (siClathrin HC) targeting siRNAs where indicated. Infectious virus yield was measured using TE671 cells as targets, and FACS analysis to enumerate infected cells. (G) Infectious HIV-1 virion production, as measured by TZM-bl indicator assay, after transfection of 293T cells with Env-defective proviral plasmids, a VSV-G expression plasmid and either an empty vector or a plasmid expressing AP180C. Data are expressed as in (D).

When introduced into GagPol expression vectors that were then used to transfect 293T cells, the class II HIV-1 IN mutations that blocked clathrin incorporation (N184L, F185K or δCTD) had only minor effects on overall particle yield (Figure 5A). However, as expected, viruses encoding these mutations exhibited extremely low infectivity (Figure 5B). More importantly, depletion of clathrin HC using siRNA did not affect overall particle yield from 293T cells (Figure 5C), but caused significant decrease in infectiousness (5 to 20-fold) of HIV-1 particles generated by cotransfection with an HIV-1 proviral plasmid (Figure 5D). Alternatively, overexpression of AP180 C-terminal domain (AP180C), which binds clathrin and induces its mislocalization [21]–[22], reduced the incorporation of clathrin into HIV-1 particles (Figure S5) and also reduced the infectiousness of HIV-1 particles generated from a proviral plasmid by >100 fold (Figure 5E) without affecting physical particle yield (Figure 5C). However, clathrin HC depletion using siRNAs, or perturbation by AP180C overexpression, had comparatively modest, but nevertheless significant, effects (∼3-fold) on the infectivity of VSV G-pseudotyped HIV-1 particles (Figure 5F and 5G). Thus, while clathrin depletion and sequestration clearly impacted Pol protein levels and, consequently, virion infectivity, there was a significant discrepancy in the magnitude of the infectivity impairment induced by clathrin perturbation when VSV-G pseudotyped HIV-1 particles were examined as compared to those generated using the natural HIV-1 envelope. Given clathrin's role in the secretory pathway, it was possible that clathrin perturbation might have effects on the HIV-1 envelope protein that impact virion infectivity, independent of its effects on Pol. In fact, generation of HIV-1 particles in the presence of AP180C resulted in virions that contained primarily unprocessed gp160 envelope protein, and very little gp120 (Figure S6). Therefore, it appears likely that clathrin affects the trafficking of the HIV-1 Env protein, or cellular factors required for Env maturation. While these results indicate caution in the interpretation of the effects of clathrin perturbation on virion infectivity, results with M-PMV (see below) suggest the clathrin perturbation using siRNA or AP180C expression does not cause a non-specific effect on the infectivity of retroviruses, and in particular the function of the VSV-G envelope protein. Thus, the effect of clathrin siRNA and AP180C on VSV-G pseudotyped HIV-1 infectivity (Figure 5F and 5G) should be independent of effects on the envelope protein.

### Clathrin incorporation and effects on SIVmac Gag

**Figure 6:**
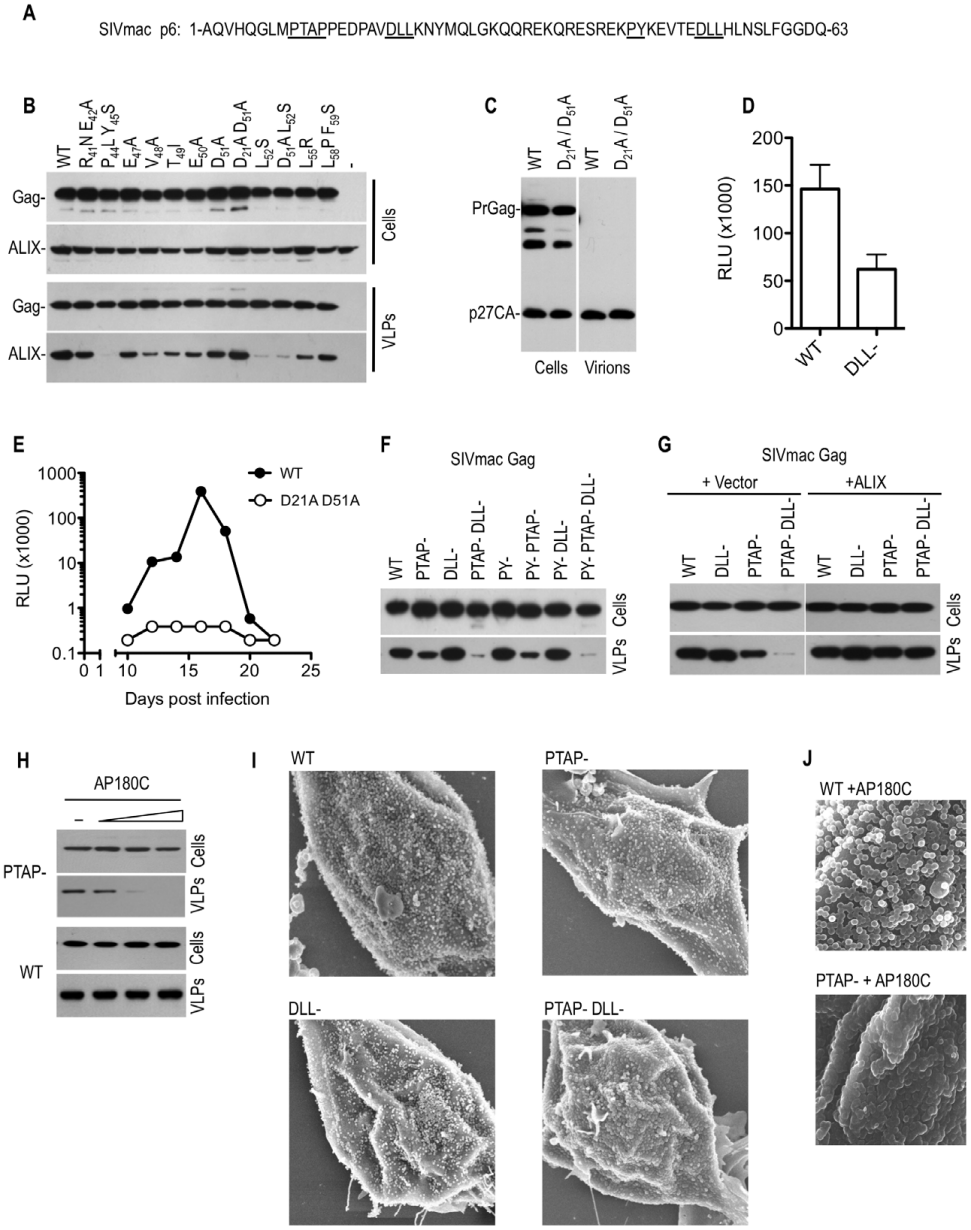
Effects of mutations in the DLL motif in SIVmac p6 on replication and particle morphogenesis. (A) Sequence of SIV p6 highlighting motifs that were mutated to specifically block recruitment of Tsg101 (PTAP), ALIX (PY) or clathrin (DLL). (B) Mapping of the ALIX-binding site on SIVmac p6. WT or mutant SIVmac Gag expression plasmids were co-transfected with a plasmid expressing HA-tagged ALIX. Cell lysates and pelleted VLPs were subjected to Western blot analysis with anti-CA and anti-HA antibodies. (C and D) Effects of DLL- mutation on SIVmac particle yield and infectiousness. WT and DLL-mutant SIVmac proviral plasmids were transfected into 293T cells. Cell lysates and pelleted VLPs were subjected to Western blot analysis with anti-CA antibody (C) and infectious virion release was quantitated using the TZM-bl assay (D). (E) Effects of DLL- mutation on SIVmac replication. C8166 cells were inoculated with equal amounts (as determined by infectious virus measurement) of WT and D_21_A/D_51_A mutant SIVmac virus. Supernatant samples were collected at 2–3 day intervals thereafter and the amount of infectious SIVmac contained therein was measured using the TZM-bl assay. (F) Effects of combined PTAP, PY and DLL-motif mutation on SIVmac Gag VLP yield. 293T cells were transfected with plasmids expressing WT or the indicated mutant SIVmac Gag proteins. Cell lysates and pelleted VLPs were subjected to Western blot analysis with anti-CA antibodies. (G) Effects of ALIX overexpression on SIVmac VLP yield. 293T cells were transfected with an empty vector or an ALIX expression plasmid along with plasmids expressing codon-optimized versions of the WT or the indicated mutant SIVmac Gag proteins. Cell lysates and pelleted VLPs were subjected to Western blot analysis with anti-CA antibodies. (H) Effects of AP180C overexpression on SIVmac VLP yield. 293T cells were transfected with an empty vector or increasing amounts (0, 0.5, 1, or 1.5 ug) of an AP180C expression plasmid along with plasmids expressing PTAP mutant or WT SIVmac Gag proteins. Cell lysates and pelleted VLPs were subjected to Western blot analysis with anti-CA antibodies. (I) Scanning EM images of 293T cells transfected with plasmids expressing codon optimized WT or the indicated mutant SIVmac Gag-IRES-eGFP cassettes. Cells were selected for imaging based on similar levels of GFP fluorescence. Additional examples are shown in Figure S7. (J) Scanning EM images of 293T cells transfected with plasmids expressing codon optimized SIVmac Gag-IRES-eGFP cassettes, in the presence of AP180C. Cells were selected for imaging based on similar levels of GFP fluorescence. Additional examples are shown in Figure S8.

In SIVmac, MLV and M-PMV Gag proteins, we noticed that motifs responsible for clathrin incorporation into virions were situated proximal to motifs responsible for recruitment of factors (ESCRT pathway-associated proteins or ubiquitin ligases) involved in viral budding (Figure 2D and 3A). In the case of SIVmac, one of the two DLL motifs is positioned overlapping the putative ALIX binding site at the C-terminus of p6 (Figure 6A). Therefore, to facilitate an examination of the role for clathrin in SIVmac replication, we first identified residues that were critical for ALIX recruitment, as well as residues that were critical for clathrin recruitment that could be mutated without disrupting ALIX recruitment. An SIVmac Gag expression plasmid was subjected to scanning mutagenesis throughout residues 41–60 of p6 (Figure 6A), with mutations selected so as not to alter the underlying p6-Pol (p6*) protein sequence. Mutant SIVmac Gag proteins were coexpressed with HA-tagged ALIX, and ALIX incorporation into VLPs was assessed by Western blot analyses (Figure 6B). Mutations P_44_L Y_45_S, L_52_S and D_51_A/L_52_S dramatically affected ALIX incorporation into VLPs, while the mutations D_21_A, D_51_A, L_58_P and F_59_S, did not (Figure 6B) These results are consistent with a recent study that also mapped the ALIX binding site in SIVmac p6 [25]. Thus, the SIVmac p6 domain was capable of recruiting clathrin and ALIX into VLPs through overlapping peptide sequences (D_21_LL D_51_LL for clathrin and P_44_Y_45_ L_52_ for ALIX), but mutants were readily identified that separately inhibited these activities. Therefore, in the ensuing studies, an SIVmac Gag protein encoding the D_21_A and D_51_A mutations in p6 was termed (DLL-) and used as a clathrin–recruitment defective mutant while the P_44_L/Y_45_S mutant was termed (PY-) (Figure 6A) and used as a ALIX-recruitment defective mutant.

To investigate the potential role of clathrin recruitment by Gag in SIVmac replication, we first compared the infectivity of particles generated by wild type and (DLL-) SIVmac proviral plasmids in a single round infectivity assay. A modest reduction (2–3 fold) in infectiousness was observed as a consequence of DLL motif mutations, with no obvious effect on physical particle yield (Figure 6C and 6D). Similar results were obtained when VSV-G pseudotyped SIVmac particles were used (unpublished observations). However, in spreading replication assays conducted in C8166 cells, this modest difference was apparently amplified, and the SIVmac (DLL-) virus was highly attenuated compared to the wild-type counterpart (Figure 6E).

Given the proximity of the DLL motifs to the L-domains in SIVmac Gag (Figure 6A), we next tested the effects of mutations predicted to abolish the recruitment of Tsg101 (PTAP-), clathrin (DLL-) and ALIX (PY-), either alone or in combination, on particle release. Initially this was done in the context of SIVmac Gag, in the absence of the viral protease (Figure 6F). Consistent with a previous report [26], mutation of the PTAP motif alone had a surprisingly modest effect on SIVmac particle yield. Moreover, mutation of the ALIX binding sites had no discernable impact on particle yield, when introduced either alone or in combination with the PTAP mutation (Figure 6F and 6G). Strikingly, while the DLL- mutation had no effect on VLP yield when introduced alone, the combination of this mutation with the PTAP- mutation dramatically diminished VLP yield (Figure 6F). This finding held true when the SIVmac Gag protein was expressed using a human codon optimized construct (Figure 6G). Overexpression of ALIX has previously been reported to rescue PTAP-mutant HIV-1 particle release [27], [28] and we found that overexpression of ALIX restored the defect in VLP release associated with the PTAP- DLL- double mutant (Figure 6G). This activity required an intact ALIX binding site, because ALIX overexpression failed to rescue the budding defect in the PTAP-DLL-PY- triple mutant (unpublished observations). This finding indicates that the PTAP-DLL- double mutant is fully competent to assemble into virions, but requires ALIX overexpression to complete budding.

Importantly we also found that overexpression of AP180C recapitulated the effect of the DLL- mutation in SIVmac Gag, and inhibited the release of VLPs assembled using PTAP- Gag (Figure 6H). AP180C overexpression did not inhibit particle release when wild-type Gag was used and this finding suggested that clathrin facilitates the completion of assembly and/or budding of SIVmac particles, particularly when budding is impaired or retarded by inhibition of Tsg101 recruitment.

To further characterize the defects in VLP release imposed by the PTAP and DLL- mutations, we analyzed 293T cells expressing wild-type and mutant SIVmac Gag proteins via scanning electronic microscopy. To eliminate variations from transfection levels, Gag expression constructs were utilized which contained an internal ribosomal entry site (IRES) linked to a GFP coding sequence on the same mRNA as Gag, and cells expressing equivalent amounts of GFP were chosen for analysis. Virions assembled using Gag proteins bearing individual PTAP- or DLL- mutations exhibited no gross morphological abnormalities, and numerous apparently spherical particles were observed on the plasma membrane of cells (Figure 6I and Figure S7). However, PTAP- DLL- double mutant Gag proteins exhibited a clear morphogenesis defect (Figure 6I and Figure S7). Specifically, cells exhibited hemispherical protrusions from their surfaces, but complete spherical particles were almost never observed. Crucially, this morphological defect could be induced using the PTAP- single mutant SIVmac Gag protein (but not the wild type Gag protein) upon overexpression of AP180C (Figure 6J and Figure S8). Together, these data strongly suggest that clathrin plays a facilitating role in the morphogenesis of SIVmac virions that is modest when measured in the context of a single cycle of SIVmac assembly, but is sufficient to strongly enhance replication, and becomes particularly evident when budding is inhibited or slowed by mutation of the PTAP L-domain.

**Figure 7:**
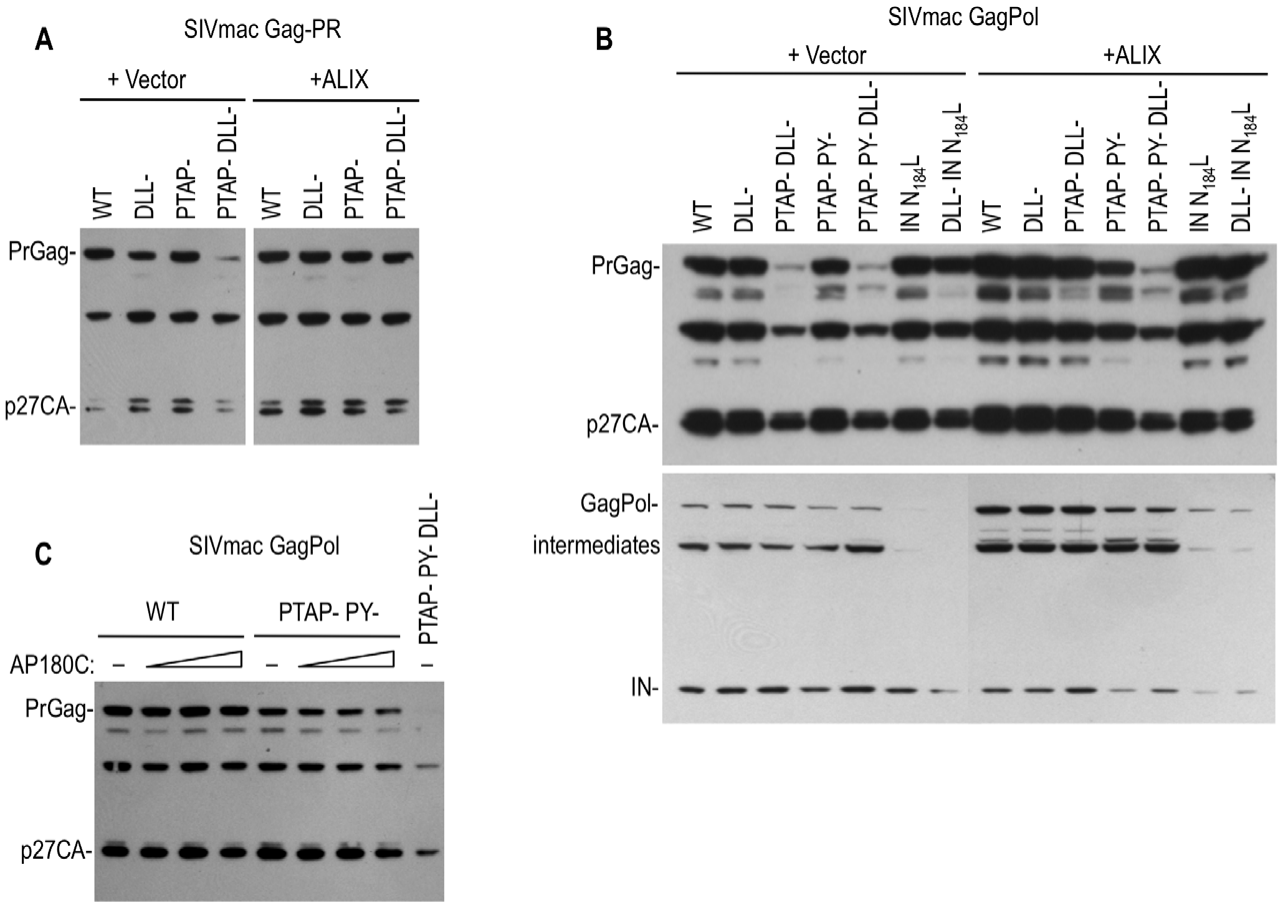
Effects of Clathrin, Tsg101 and ALIX on the levels of SIVmac Gag and Pol proteins. (A) Effects of DLL motif, PTAP and PY mutations and ALIX overexpression on the expression of SIVmac Gag protein in the presence of an active viral protease. 293T cells were transfected with WT or the indicated mutant SIVmac GagProtease expression plasmids along with an empty vector or an ALIX expression plasmid. Cell lysates were subjected to Western blot analysis with an anti-CA antibody. (B) Effects of DLL motif, PTAP, PY and IN mutations, and ALIX overexpression on the expression of SIVmac Gag and Pol proteins. 293T cells were transfected with WT or the indicated mutant SIVmac GagPol-HA expression plasmids along with an empty vector an ALIX expression plasmid. Cell lysates were subjected to Western blot analysis with an anti-CA (upper panel) or anti HA (lower panel) antibodies. (C) Effects of PTAP mutation and AP180C overexpression on the expression of SIVmac Gag protein in the context of GagPol. 293T cells were transfected with WT or the indicated mutant SIVmac GagPol-HA expression plasmids along with an empty vector or 250, 500 or 1000 ng of AP180C expression plasmid. Cell lysates were subjected to Western blot analysis with an anti-CA antibody.

When SIVmac Gag proteins were expressed in the context of an active viral protease, either using GagProtease (Figure 7A) or full-length GagPol (Figure 7B) expression plasmids, the DLL- single mutation had little or no effect on levels of cell associated Gag protein (Figure 7A and 7B). However, when the DLL- mutation was present in combination with the PTAP- mutation, the steady state cell-associated levels of the Gag precursor and processed derivatives were significantly diminished (Figure 7A and 7B). Remarkably, overexpression of ALIX rescued this defect and restored the level of PTAP- DLL- mutant SIVmac Gag proteins to those expressed by the wild type Gag-protease and GagPol expression plasmid (Figure 7A and 7B). Notably, the ability of ALIX to restore PTAP- DLL- mutant SIVmac Gag protein levels required an ALIX binding site, as this effect was not observed when a PTAP- DLL- PY- mutant SIVmac GagPol expression plasmid was used (Figure 7B). The effect of the DLL- mutation on SIVmac Gag expression levels was partially recapitulated by clathrin sequestration using AP180C. Specifically, AP180C overexpression caused a reduction in the levels of Gag protein expressed by a PTAP- PY- mutant, but not wild-type, GagPol expression plasmid (Figure 7C). Taken together, these findings lead to the conclusion that clathrin interaction with SIVmac Gag facilitates virion morphogenesis, with consequent effects on Gag protein stability in the presence of the viral protease, especially when budding is impaired.

The effects of the DLL- mutation on SIVmac Gag protein levels were specific to Gag, and did not affect Pol protein levels (Figure 7B). Similarly, a mutation in SIVmac Pol (analogous to the HIV-1 IN N184L) that blocked Pol dependent clathrin incorporation (Figure 2G) caused a reduction in the level of SIVmac Pol protein to an almost undetectable level, while Gag levels were unaffected (Figure 7B). In contrast to the effect of ALIX on SIVmac Gag expression, this Pol expression defect could not be rescued by overexpression of ALIX. Thus, the effects of mutations that reduce protease-dependent Gag stability and clathrin incorporation were independent of those that affected Pol stability and clathrin incorporation.

### Effects of clathrin recruitment on MLV infectivity and Gag proteins

**Figure 8:**
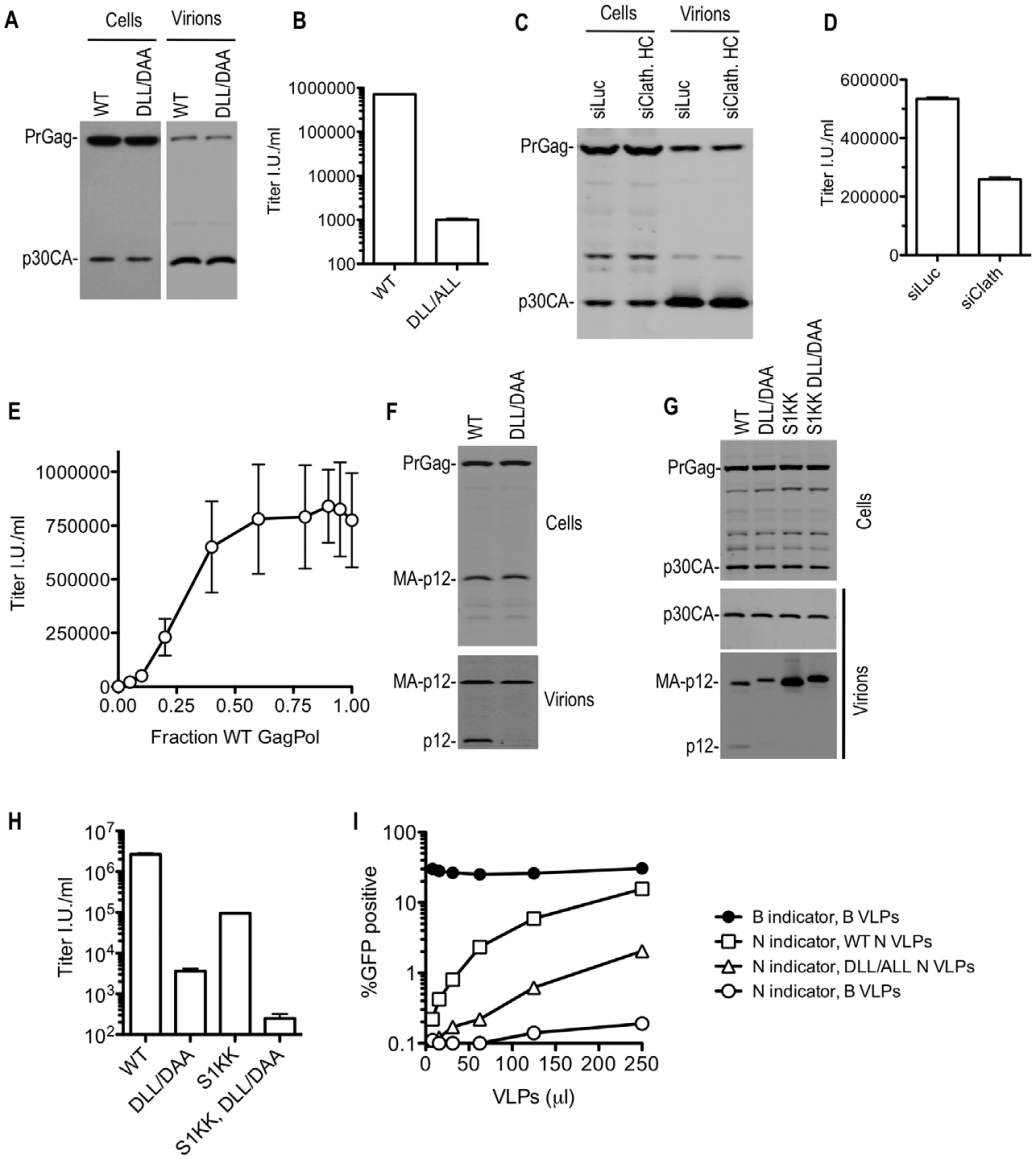
Disruption of clathrin incorporation into MLV particles is associated with impaired infectivity. (A and B) Loss of infectivity of DLL- mutant MLV particles. 293T cells were transfected with wild-type or DLL-mutant MLV GagPol expression plasmids, along with a VSV-G expression plasmid and a GFP-expressing MLV vector. Cell lysates were subjected to Western blot analysis with an anti-MLV CA antibody (A). TE671 cells were inoculated with the supernatant of these cultures and the number of infected cells enumerated by FACS analysis (B). (C and D) Effect of clathrin depletion on MLV infectivity. 293T cells were transfected as in (A and B) except that cells were also transfected with luciferase (siLuc) or clathrin HC (siClathrin HC) targeting siRNAs where indicated. Western blotting analysis (C) and infectious virion measurements (D) were carried out as in (A and B). (E) Small amounts of coexpressed WT MLV GagPol suppress the infectivity defect exhibited by DLL-mutant MLV GagPol. Varying amounts (0, 5, 10, 20, 40, 60, 80, 90, 95, or 100 ng) of wild-type (WT) GagPol expression plasmid were co-transfected with varying amounts (100, 95, 90, 80, 60, 40, 20, 10, 5, or 0 ng) of a DLL-mutant MLV GagPol expression plasmid along with a VSV-G and GFP-expressing MLV vector plasmid. Infectious virion yield was measured using TE671 cells and plotted as a function of the fraction of the GagPol plasmid that was WT. Mean values ±standard deviations are shown. (F) Absence of intact p12 in DLL mutant MLV VLPs. Cell lysates and virions generated in (A) were subjected to Western blot analysis with an anti-p12 antibody. (G and H) Absence of mature p12 in virions is symptomatic but not the sole underlying cause of reduced infectiousness of DLL-mutant MLV virions. 293T cells were transfected with wild-type or the indicated mutant MLV GagPol expression plasmids, along with a VSV-G expression plasmid and a GFP-expressing MLV vector. Western blotting analysis (G) and infectious virion measurements (H) were carried out as in (A and B). (I) DLL-mutant MLV VLPs exhibit reduced ability to saturate TRIM5α. TE671 cells were inoculated with a fixed dose of GFP-expressing MLV vectors packaged using N-MLV and B-MLV GagPol proteins in the presence of increasing amounts of wild-type or DLL- mutant N-MLV VLPs, or B-MLV VLPs. The percentage of infected (GFP-positive) cells is plotted as a function of VLP dose.

Like SIVmac, MLV has a DLL motif proximal to its L-domain that is responsible for clathrin incorporation (Figure 3A). Transfection of an MLV GagPol expression plasmid in which the DLL motif was mutated to either ALL or DAA, along with plasmids encoding VSV-G and a GFP expressing MLV vector resulted in no defect in the yield of physical particles (Figure 8A), but mutant particles were >100-fold less infectious than WT particles (Figure 8B). Similar results were obtained using full-length MLV proviral plasmids (unpublished observations). Similarly, siRNA mediated clathrin depletion did not affect particle yield (Figure 8C). However, clathrin depletion resulted in only a modest reduction of MLV infectivity (Figure 8D). This may be attributable to incomplete clathrin knockdown (approximately 20% of normal clathrin levels remained in siRNA transfected cells, Figure S3A). Consistent with this notion, coexpression of WT and DLL- mutant MLV GagPol at various ratios revealed that particles were nearly fully infectious, even when a small fraction of the Gag proteins contained therein harbored an intact DLL motif (Figure 8E). Therefore, we hypothesize that the residual clathrin recruited by WT virus assembled in siRNA treated cells may be nearly sufficient to fulfill its functional role.

Western blot analysis showed that substitutions in the MLV DLL motif had no effect on cell- or extracellular virion- associated levels of viral precursor Gag (Pr65) or processed p30 CA proteins when expressed in the context of MLV GagPol or proviral plasmids (Figure 8A and unpublished observations). This held true when the DLL mutation was introduced alone, or in combination with mutations in PPPY and/or PSAP motifs (unpublished observations). However, Western blot analysis with an anti-p12 monoclonal antibody revealed a dramatic decrease in the levels of the mature p12 protein associated with virions (Figure 8F). Conversely the partly processed MA-p12 intermediate was present at equivalent abundance in wild type and mutant virions. This finding excluded the possibility that the p12 antibody failed to recognize the DAA mutant p12 sequence and instead suggested the possibilities that either the MA-p12 junction was not efficiently processed in the DAA mutant, or that the DAA mutation created a p12 that was aberrantly cleaved and as a consequence could not be recognized by the p12 antibody. To investigate these possibilities, a small amount of wild-type or DLL-mutant MLV Gag-Pol expression plasmid was co-transfected with increasing amounts of wild-type or mutant Gag expression plasmids (in a Gag-Pol ∶ Gag ratio of 1∶0.5 to 1∶16, (Figure S9)). This analysis suggested that the DAA mutant and WT Gag proteins could be processed in *trans* by coexpressed GagPol bearing the WT p12 sequence, yielding mature p12 protein in extracellular virions with comparable efficiency. Conversely, the DAA mutant GagPol protein failed to generate the fully processed p12 in virions, even when a WT Gag protein was provided in *trans* (Figure S9). In a similar experiment, we used a different MLV-related GagPol expression plasmid (from XMRV) whose p12 sequence is poorly recognized by the anti MLV p12 antibody (Figure S10). When XMRV GagPol was coexpressed with WT or DLL mutant MLV Gag proteins, the WT and DLL mutant MLV p12 proteins were detected at approximately equivalent levels in the resulting virions (Figure S10). Thus, both the WT and mutant MLV MA-p12 junction can be efficiently processed by MLV or XMRV protease in *trans* resulting in the appearance of the mature p12 protein in virions. In other words, it appeared that the DLL motif regulates the activity of the MLV protease in *cis*, but not in *trans*, and the absence of the DLL motif in *cis* to the protease caused aberrant Gag processing and absence of p12 in virions.

While the absence of the p12 protein in DLL-mutant MLV virions appeared symptomatic of the defect induced by the clathrin binding site mutation, it was not clear whether this lesion was the direct cause of the infectivity defect. To test this, we generated MLV particles using a construct, similar to one previously described [29], in which the MA-p12 cleavage site was mutated (S1KK) so that it could not be cleaved, resulting in the absence of the mature p12 protein in virions (Figure 8G). In this context, the DLL- mutation retained its deleterious effect on MLV infectivity (Figure 8H). Thus, these findings suggested the existence of a generalized morphological defect that included, but was not limited to, p12 deficiency in MLV particles consequent to mutation of the clathrin binding site.

Electron microscopic analysis of WT and DLL mutant MLV expressing cells did not reveal any gross morphological alterations in assembling or assembled particles (Figure S11A). Therefore, to test for more subtle morphogenesis defects, we examined whether DLL- mutant particles retained the ability to saturate the TRIM5α restriction factor. This assay should test for the presence of a stable, morphologically accurate capsid lattice, which is expected to be required for efficient binding to, and saturation of TRIM5α. Thus, increasing amounts of N-tropic MLV particles generated using WT or DLL- mutant GagPol expression plasmids (Figure S11B) were applied to human TE671 cells, which express a TRIM5α protein that can recognize and restrict N-tropic MLV capsids [30], [31]. Simultaneously, a fixed and equal amount of GFP-expressing WT B-tropic or N-tropic indicator virus was applied to monitor TRIM5α saturation (Figure 8I). Wild-type N-tropic MLV particles efficiently saturated TRIM5α and thereby facilitated N-tropic MLV infection. However, particles containing a mutation in the DLL motif in p12 were approximately 10-fold less active in this TRIM5α saturation assay (Figure 8I and S11B). The finding that the mutant virions were less ‘visible’ to human TRIM5α suggests that their cores were not properly formed, or unstable and, therefore, that the clathrin-binding motif in p12 is important for accurate MLV particle morphogenesis.

### Effect of clathrin on the infectiousness of M-PMV virions

Like SIVmac and MLV, the betaretrovirus M-PMV harbours a motif (DLISLD) proximal to its L-domains that is responsible for recruiting clathrin into virions. Notably, however, the morphogenesis pathway for M-PMV is quite different to that of SIVmac and MLV, in that complete immature capsids are assembled in the cytoplasm and move thereafter to the plasma membrane for envelopment.

**Figure 9:**
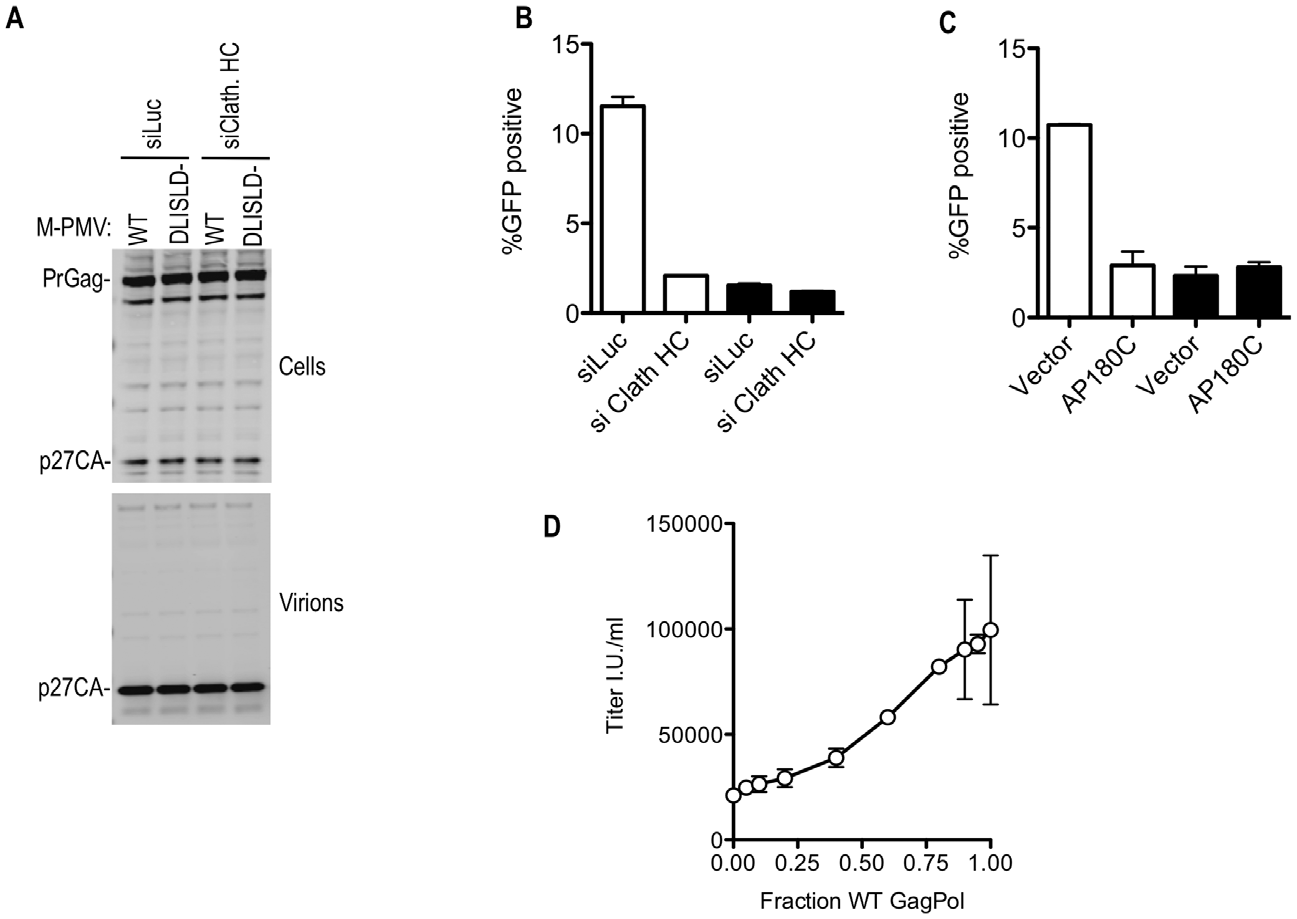
Effects of clathrin on M-PMV Infectivity. (A) No effect of clathrin binding site mutation or clathrin depletion on M-PMV particle yield. 293T cells were transfected with wild-type or DLISLD mutant M-PMV proviral plasmids, along with a VSV-G expression plasmid. Cells were simultaneously transfected with luciferase (siLuc) or clathrin HC (siClath. HC) targeting siRNAs where indicated Cell lysates were subjected to Western blot analysis with an anti-M-PMV CA antibody. (B) The DLISLD mutation inhibits M-PMV infectivity and knockdown of clathrin by siRNA inhibits WT but not DLISLD mutant M-PMV infectivity. 293T cells were transfected with wild-type (white bars) or DLISLD mutant (black bars) GFP-expressing M-PMV proviral plasmids (pSARM-X-GFP) along with a VSV-G expression plasmid, as in (A) along with luciferase (siLuc) or clathrin HC (siClath. HC) targeting siRNAs where indicated. The yield of infectious virions in the culture supernatant was measured by FACS after inoculation of TE671 cells. (C) AP180C overexpression inhibits WT but not DLISLD mutant M-MPV infectivity. 293T cells were transfected as in (B) except that the siRNAs were replaced with an empty plasmid vector or an AP180C expression plasmid. The yield of infectious virions in the culture supernatant was measured as in (B). (D) Varying amounts (0, 10, 20, 40, 80, 120, 160, 180, 190, or 200 ng) of wild-type (WT) GFP-expressing M-PMV proviral plasmid was co-transfected with varying amounts (200, 190, 180, 160, 120, 80, 40, 20, 10, or 0 ng) of a DLISLD mutant plasmid and a VSV G expression plasmid. The yield of infectious virions was measured on TE671 cells and plotted as a function of the fraction of the proviral plasmid that was WT. Mean values ±standard deviations (SDs; bars) are shown.

Mutation of the clathrin-recruiting motif in M-PMV Gag reduced the infectiousness of M-PMV virions by 5-fold, without affecting the yield of physical particles (Figure 9A and 9B) or causing gross morphological abnormalities that could be visualized by electron microscopic examination (unpublished observations). Notably, clathrin depletion using siRNA or perturbation by overexpression of AP180C had a negative effect on M-PMV infectivity of similar magnitude to that of the clathrin binding site mutation (Figure 9B and 9C). Importantly, however, the effect of depletion or sequestration of clathrin on M-PMV infectivity was specific to the WT virus; these manipulations had no effect on the infectivity of the mutant M-MPV that could not recruit clathrin (Figure 9B and 9C), suggesting that the clathrin binding site mutation and the clathrin perturbation affected the same process.

It was surprising that the clathrin knockdown had a greater effect on M-MPV infectivity compared to MLV infectivity (compare Figure 8D and 9B), while M-PMV was less affected by clathrin binding site mutations than was MLV (compare Figure 8B and 9B). However, experiments in which M-MPV virions were generated using mixtures of WT and DLISLD mutant proviral plasmids showed that reduction in infectivity was approximately proportional to the fraction of the Gag protein that was mutant versus WT (Figure 9D). Thus, M-MPV infectivity appeared more sensitive to partial depletion of clathrin, or partial removal of clathrin binding sites from virions, than did MLV. Nonetheless, optimal infectiousness and replication of both viruses was clearly dependent on clathrin recruitment by their respective Gag proteins.

## Discussion

In this study, we identified clathrin as a component in a variety of retrovirus particles, including members of the lentivirus (HIV-1 and SIVmac), gammaretrovirus (MLV) and betaretrovirus (M-PMV) genera. Specifically, the Gag proteins of SIVmac, MLV and M-PMV recruit clathrin HC using DLL or DLISLD motifs, which mimic those commonly found in classical clathrin adaptors. Additionally, HIV-1 Pol and, to some extent, SIVmac Pol were capable of recruiting clathrin into virions, although the motifs responsible could not be mapped because clathrin incorporation appeared to be dependent on the conformational integrity of multiple domains of the HIV-1 Pol protein.

Phenotypic characterization of mutant viruses and a combination of other techniques to perturb clathrin in virus-producing cells (siRNA-based clathrin HC knockdown or AP180C overexpression), revealed that clathrin can have a range of apparently distinct effects on virion morphogenesis, depending on the particular retrovirus examined. These effects are summarized in Table S2. In the case of HIV-1, mutations or drugs (efavirenz) that affect RT dimerization as well as class II IN mutations are known or thought to affect protease activation [17], [23], [24], [32], [33], [34], [35]. We found that at least some of these mutations blocked clathrin incorporation, and also found that clathrin overexpression could ameliorate deficits in the levels of Pol protein harboring some of the aforementioned mutations. Moreover, depletion or sequestration of clathrin could reduce the levels of Pol proteins in cells. This suggests that clathrin may be directly involved in regulating protease activity, or perhaps in stabilizing or retaining the products of Pol proteolytic cleavage during HIV-1 morphogenesis.

Similarly, mutations in the clathrin-recruiting motif in the p6 domain of SIVmac Gag, as well as sequestration of endogenous clathrin, decreased the level of the viral Gag protein in the presence of the viral protease. However, these effects were only truly evident when the viral PTAP motif was also mutated. Again this finding is consistent with the notion that clathrin regulates proteolysis of the viral protein, or stabilizes the products of proteolysis, and the magnitude of the effect is exaggerated to easily detectable levels if budding is blocked or retarded. Notably, the apparent protease-dependent instability of the PTAP- DLL- mutant Gag protein could be largely reversed by overexpression of ALIX, strongly suggesting that the PTAP- DLL- mutant Gag protein is not generically unstable or acutely sensitive to proteolysis simply as a direct consequence of the introduced mutations. Rather, this observation reinforces the notion that protease-dependent SIVmac Gag depletion, consequent to a failure to recruit clathrin, is exaggerated by retarding the rate of particle budding.

In the case of MLV, there was no apparent effect of the DLL- mutation on Gag precursor stability, even when the proximal PPXY L-domain motif was mutated. However, there was a very clear effect on the products of Gag proteolysis, in that p12 was absent from virions. Moreover, the DLL mutation had major effects on the infectivity of MLV particles, as well as the accuracy of their morphogenesis, or their stability, as evidenced by the relative inefficiency with which MLV cores were apparently recognized by the TRIM5α restriction factor. However the precise nature of the biochemical lesion responsible for this infectivity defect remains to be completely defined. The absence of p12 from virions could contribute to the infectivity defect, as p12 has been shown to be a component of MLV preintegration complexes [36]. However, mutations that prevent the cleavage of p12 from MA did not abolish the effect of the DLL-mutation on MLV infectivity, suggesting additional effects of clathrin on particle morphogenesis.

In addition to effects on viral protein proteolysis, clathrin appears to affect the morphogenesis and release of SIVmac particles in the absence of the viral protease. Again, observing this effect required the PTAP-motif to be mutated, and the effect could be suppressed by overexpression of ALIX. The ability of ALIX overexpression to suppress the two effects of clathrin binding site mutation on SIVmac, namely; (i) an assembly defect in the absence of the viral protease and (ii) Gag instability in the presence of the viral protease, suggests that they are different manifestations of the same defect in clathrin recruitment. This apparent ability to affect both protease-dependent and protease-independent processes influences the generalized models that can be invoked to explain the role of clathrin in retrovirus morphogenesis (see below).

It is interesting that the D_51_L_52_L_53_ motif in SIVmac Gag overlaps with the ALIX binding site, raising the possibility that ALIX and clathrin might compete with each other. Moreover, inspection of multiple viral strains of the SIVsm/SIVmac/HIV-2 lineage reveals that all p6 sequences contain 1, 2 or 3 copies of a DLL motif. In those that contain a single DLL motif, it appears that one copy has been displaced by a PTAP motif. These observations are suggestive of interplay between clathrin and the ESCRT machinery that merits future investigation.

A caveat to the aforementioned conclusions is that mutations, particularly in HIV-1 or SIVmac Pol might have pleiotropic effects, and it therefore was difficult to assign cause and effect in situations where, for example, Pol mutations both blocked clathrin incorporation and caused decreases in the levels of Pol proteins. Nonetheless, depletion or sequestration of clathrin could, in several cases, at least partly recapitulate the effects of mutations that blocked clathrin recruitment. The pleiotropic effect of HIV-1 Pol mutations likely arises from the fact that they cause premature protease activation, and because protease has multiple substrates in Gag and Pol, a variety of effects on the accuracy of particle assembly and Pol protein incorporation, and therefore particle infectiousness, are predictable consequences. It is possible that the pleiotropic effect of these mutations is a consequence of their effects on clathrin recruitment. Indeed, overexpression of clathrin, or fusion of a clathrin binding site in *cis* could increase the levels of HIV-1 Pol proteins that were apparently destabilized by Pol mutations. However, it was not possible to restore the infectiousness of Pol-mutant particles using this approach.

One factor that must be considered in arriving at a proposal for general model for a role of clathrin in retroviral replication was that the magnitude of the effect on infectious virion yield clearly varied according to which retrovirus was examined. Additionally, clathrin was not found to be efficiently incorporated into all retroviruses that we tested. For instance, equine infectious anemia virus (EIAV) and human endogenous retrovirus K (HERV-K) did not efficiently incorporate clathrin (unpublished observations). Moreover, the apparent effect of clathrin on retrovirus replication usually varied according to whether the experimental manipulation was clathrin binding site mutation, clathrin depletion, or clathrin sequestration. In some cases (e.g. SIVmac Gag), the effect of mutations in defined motifs responsible for clathrin recruitment was relatively modest in a single cycle of replication, while in others (e.g. MLV) the effect was dramatic. In the case of HIV-1, SIVmac and MLV the effects of mutations that abolish clathrin incorporation were much greater than the effects of clathrin depletion or sequestration. While this may be due to pleiotropic effects of the mutations that were introduced, it is also true that clathrin depletion or sequestration using the techniques employed herein was incomplete and, at least in the case of MLV, a relatively small fraction of the viral Gag protein is needed to be capable of recruiting clathrin in order for virions to be nearly fully infectious. Conversely, in the case of M-PMV, the magnitude of the effect of clathrin binding site mutation on particle infectivity was nearly precisely recapitulated by clathrin depletion or sequestration. Moreover, the impaired infectivity of the clathrin recruitment-defective mutant was not further impaired reduced by clathrin depletion or sequestration. Thus, we can be quite confident in the case of M-PMV case that clathrin enhances infectivity primarily through the action of the DLISLD motif in Gag. Details of the mechanism by which clathrin enhances M-PMV infection remain to be investigated and are difficult at present due to the paucity of reagents for studying this virus, but M-PMV may make the most tractable system for future investigations.

It is challenging, based on these data, to make definitive general conclusions as to the role of clathrin in retrovirus replication, because the ultimate outcome, in terms of defined lesions that occurred as a consequence of blocking clathrin recruitment, differed in each retrovirus studied herein (Table S2). Moreover, in some retroviruses Pol was responsible for clathrin recruitment, while in others Gag was responsible. Although conceptually unsatisfying, it may simply be the case that clathrin plays a completely different role in each retrovirus. However, for HIV-1, SIVmac and MLV, a common theme was that clathrin appeared to influence proteolysis of the viral polyproteins. This would likely have different, potentially pleiotropic, consequences for different retroviruses, including infectivity and morphogenesis defects as well as apparent viral protein instability. We observed all of these outcomes in this study and each could be reasonably hypothesized to be the consequence of mis-regulation of viral polyprotein processing.

Models that could potentially explain the aforementioned phenomena include the idea that clathrin contributes to the spatial organization of Gag and Pol proteins during particle assembly. As a homotrimeric Gag or Pol binding protein, clathrin could bind to multiple Gag or GagPol molecules simultaneously. Indeed, the affinity of clathrin for cellular clathrin adaptors, and likely, therefore, assembling Gag or Pol proteins, is dependent on their multimerization/polyvalency [12], [13]. The association of clathrin with assembling Gag and Pol proteins could influence proteolysis of the viral proteins by positively or negatively influencing protease dimerization (which is required for protease activity), by influencing substrate (Gag or Pol) mobility and/or accessibility to the protease, or by helping to retain or remove the products of partial or complete proteolysis from a nascent virion as particle assembly and proteolysis proceeds. Such a model can also be reconciled with the findings that the effects of clathrin appear quite variable, from overt effects on particle morphogenesis and viral protein stability to more subtle effects on virion infectiousness.

## Materials and Methods

### Plasmids and construction

The proviral HIV-1 plasmid used throughout was pNL4-3 (NIH AIDS Research and Reference Reagent Program, Catalog No. 114). A protease defective variant, and other mutants thereof, were constructed by generating PCR products harboring mutations in PR or RT or IN, that were digested with SphI and EcoRI before subcloning. The SIVmac proviral plasmid was previously described [37] and based on SIVmac239. An MLV proviral plasmid (pNCS) was a gift from Stephen Goff and the M-PMV proviral plasmid pSARM-4 was a gift from Eric Hunter.

Overlap-extension PCR was used to generate HIV-1, SIVmac, MLV, and MPMV mutants. For HIV-1 Gag, HIV-1 GagPol, SIVmac Gag, SIVmac GagProtease (Gag-PR) and SIVmac GagPol expression plasmids, wild-type or mutant encoding sequences were amplified by PCR, using primers that incorporated 5′EcoRI and 3′ NotI sites and inserted into the HIV-1-based expression plasmid pCRV1 [38]. In some cases an HA epitope was inserted, fused in frame at the C-terminus of the expressed protein into pCRV1 vector. The panel of SIVmac double mutants PTAP- (PTAP/LTAL) DLL-(DLL/ALL), PTAP- (PTAP/LTAL) PY-(PY/LS) and DLL-(DLL/ALL) IN-N184L and triple mutants PTAP- (PTAP/LTAL) PY-(PY/LS) DLL- (DLL/ALL) were constructed by introducing additional point-mutation into pre-constructed single or double mutants, respectively. To generate chimeric HIV-1/SIVmac Gag proteins (Figure 2B), overlap-extension PCR was performed with primers targeting the corresponding region of SIVmac or HIV-1 Gag and PCR products were inserted along with a C-terminal HA-epitope tag into pCRV1. To insert mutant SIVmac p6 at the C-terminus of mutant HIV-1 GagPol, NotI sites were incorporated at both 5′ and 3′ end of the SIVmac p6-encoding PCR product which was inserted between HIV-1 Gag-Pol and the HA epitope in pCRV1.

Plasmids expressing codon-optimized SIVmac Gag, pCR3.1/SIVmac-Gag, as well as codon-optimized HIV-1 Gag, codon-optimized HIV-1 Gag-Pol and codon optimized GagGFP, were previously described [38] and mutations were made by overlap-extension PCR. To construct pCR3.1/SIVmacGag-IRES-eGFP expressing both SIVmac Gag and GFP, the IRES-eGFP region was amplified, using pIRES2-EGFP(Clontech) as template, digested by NotI and subcloned at the C-terminus of SIVmac Gag in WT and mutant pCR3.1/SIVmac-Gag expression plasmids.

For MLV mutants, PCR products harboring D_156_LL- mutations (DLL/ALL or DLL/DAA) were digested using BsrGI and XhoI and inserted into pCAGGS-based MLV Gag or Gag-Pol expression vectors [39] or an N-tropic MLV GagPol expression plasmid [30]. For M-PMV mutants, PCR products bearing the DL_129_I_130_SLD/DAASLD mutation were digested by SmaI/SacI and inserted into pSARM-4 [40] or pSARM-X-eGFP as described in [41].

Plasmids expressing clathrin HC residues 1–363aa or 1–494aa with a C-terminal HA or Myc epitope were amplified by using full-length clathrin HC as a template and inserted into pCR3.1 (Invitrogen).

The plasmid expressing pCR3.1/ALIX, the HIV-1 proviral plasmid bearing YFP in the stalk region of matrix (pNL4-3 MA-YFP) and the XMRV (xenotropic murine leukemia-related virus) GagPol expression plasmid were described previously [42], [43], [44]. The plasmid expressing FLAG-AP180C was a gift from Lois Greene, a plasmid expressing DsRed-clathrin LC was a gift from Sanford Simon and pSIV-T1 was from Francois-Loic Cosset [45].

### Antibodies

Monoclonal antibodies included anti-HA (Covance), anti- Clathrin HC (BD Transduction laboratories), anti-FLAG (Sigma), anti-HIV capsid p24 (183-H12-5C), anti-Env (1D6), anti-RT (MAb21) (all from NIH AIDS Research and Reference Reagent Program), anti-HIV IN (a gift from Michael Malim), anti-MLV capsid p30 (ATCC CRL-1912 R187), anti-MLV p12 (ATCC, CRL-1890 548), and anti-M-PMV Gag (a gift from Eric Hunter) [46]. In addition, secondary antibodies included goat anti-mouse or anti rabbit IgG conjugated to horseradish peroxidase, or to Alexa Fluor 488 (Invitrogen), IRDye® 800CW and IRDye® 680 (LI-COR Biosciences).

### Cell lines and transfection

Adherent cell lines from human (293T, TE671, TZM-bl) were maintained in DMEM supplemented with 10% fetal calf serum and gentamycin. CD4+lymphoid cell lines CEMx174, MT2, MT4 and C8166 were grown in RPMI/10%FCS/antibiotics.

In general, for transfection experiments in 293T cells, cells were seeded at a concentration of 1.5×10^5^ cells/well (24-well) or 3×10^5^ cells/well (12-well) or 2×10^6^(10-cm dish) and transfected the following day using polyethylenimine (PolySciences).

To identify proteins incorporated into HIV-1 VLPs, 10 µg of an HIV-1 GagPol and Gag expression plasmids were transfected into 293T cells in 10 cm dishes. Particles in 30 ml of filtered supernatant were pelleted through 20% sucrose, resuspended in PBS, treated with subtilisin (Sigma) and separated on Optiprep gradients. Particulate material in each of 8 fractions was recovered by diluting the fractions with PBS and ultracentrifugation and analyzed by SDS-PAGE followed by Coomassie blue or silver staining. Bands of interest were excised and protein identification was done by the Rockefeller University Proteomics Center. To determine domains responsible for clathrin incorporation, 293T cells were transfected with 5 µg of HIV-1 or SIVmac GagPol or Gag expression plasmid along with 3 µg of clathrin heavy chain 1–494aa or clathrin heavy chain 1–363aa or 0.5 µg GFP-HA expression plasmid. Analysis of clathrin incorporation into other viruses was performed by transfection of 8 µg of MLV GagPol expression plasmid or 8 µg of M-PMV proviral plasmid, with or without 2 µg of clathrin HC 1–494aa or 2 µg of clathrin HC 1–363aa expression plasmids followed by ultracentrifugation and subtilisin treatment of pelleted virions.

To measure SIVmac VLP release, cells were transfected with 150 ng of native sequence or codon-optimized SIVmac Gag expression plasmids, along with 300 ng of ALIX expression plasmid where indicated. In Figure 6H, 500 ng of codon optimized SIVmac Gag was cotransfected with 0 ng, 0.5 µg, 1.0 µg or 1.5 µg of AP180C expression vector. VLPs were harvested at 24 hrs post transfection, pelleted through 20% sucrose and subjected to Western blot analysis.

To define the ALIX binding motif on SIVmac Gag, 500 ng of plasmids expressing wild-type or mutant SIVmac Gag proteins were cotransfected with 500 ng of pCR3.1/HA-ALIX into 293T cells. Cell lysates and VLPs were harvested and analyzed by Western blotting after 40 hrs.

To generate infectious viruses bearing a GFP reporter, 293T cells were transfected with 200 ng of an HIV-1 GagPol expression plasmid and 200 ng of reporter vector pHRSIN-CSGW [47] (for HIV-1), 200 ng of an SIVmac GagPol expression plasmid and 200 ng of reporter vector pSIV-T1 (for SIVmac), 200 ng of an MLV GagPol expression plasmid and 200 ng of reporter vector pCNCG [30] (for MLV), or 400 ng of pSARM-X-eGFP (for M-PMV), along with 100 ng of plasmid expressing the VSV-G envelope. To measure the effect of AP180C on infectiousness of viruses, 500 ng of empty vector or AP180C expression plasmid was added to the transfection mixture. To generate the VSV-G pseudotyped noninfectious VLPs used in the TRIM5 saturation assays in Figure 8I and Figure S11B, 10 µg of WT or DLL- mutant N-tropic MLV and 2 µg of VSV-G expression plasmid were used to transfect a 10 cm dish of 293T cells.

To determine the effects of AP180C on HIV-1 or SIVmac viral protein expression level, 200 ng of HIV-1 proviral plasmid was cotransfected with 0 ng, 300 ng, 600 ng, 900 ng of AP180C expression plasmid (Figure 4F), or 200 ng of SIVmac GagPol expression plasmid was cotransfected with 0 ng, 250 ng, 500 ng or 1000 ng of AP180C expression plasmid (Figure 7C).

To assess the ability of the MLV protease to process WT or mutant Gag proteins (Figure S9), 50 ng of WT or DLL/DAA mutant MLV GagPol expression plasmid were contransfected with increasing amounts (0, 25 ng, 50 ng, 100 ng, 200 ng, 400 ng, 800 ng) of plasmid expressing WT or DLL/DAA mutant MLV Gag. Alternatively, in Figure S10, 6 µg of XMRV GagPol expression plasmid were cotransfected with 300 ng of WT or DLL/DAA MLV Gag expression plasmid. Cell lysates and VLPs were analyzed by Western blotting 48 hrs later.

In all transfection experiments, the total amount of DNA was held constant within the experiment by supplementing transfection mixtures where necessary with empty expression vector.

### Infectivity assays

Transfected 293T cells were placed in fresh medium at 20 hrs post infection and virion containing cell supernatants were harvested and filtered (0.22 µm) at 40 hrs post transfection. Infectious virus release (for HIV-1 and SIVmac proviral plasmid derived virus) was determined by inoculating TZM cells seeded in 96 well plates at 1.2×10^4^ cells/well. At 48 hrs post infection, β-galactosidase activity was determined using GalactoStar reagent as per the manufacturer's instructions.

Reporter viruses (HIV-1, SIVmac, Moloney MLV and M-PMV) bearing a GFP indicator gene were generated by transient transfection of 293T cells along with VSV G envelope protein, in the absence or presence of AP180C as indicated above. TE671 cells were seeded one day prior to infection, inoculated with GFP reporter viruses and FACS analysis was carried out using Guava EasyCyte instrument (Guava Technologies).

### TRIM5α saturation assays

TE671 were seeded at 2×10^4^ cells/well in 24-well plates one day before infection. Cells were inoculated with a fixed dose of N-MLV or B-MLV GFP reporter virus, selected so that infection with the restricted virus in the absence of VLPs gave low, but measurable, levels of infection (about 0.1% GFP-positive cells). Restriction-abrogating VLPs were serially diluted two-fold and added to the target cells simultaneously with the fixed dose of N-MLV in the presence of polybrene. Infection by the GFP reporter virus was measured 48 h later as described above.

### RNAi

293T cells were transfected with 60–100 pmol of a clathrin-specific RNA duplex (SMART pool, Dharmacon) or a control firefly luciferase duplex (Dharmacon) using Lipofectamine2000 (Invitrogen) according to manufacturer's instructions. Twenty-four hours post-transfection cells were harvested and replated. Forty-eight hours after the first transfection, cells were co-transfected with siRNA (as above) and 200 ng of proviral plasmid or, in the case of GFP-reporter viruses, 200 ng of GagPol expression plasmid, 200 ng of corresponding retroviral reporter plasmid along with 100 ng of VSV-G expression plasmid using Lipofectamine2000 (Invitrogen). Virions and cells were harvested 48 hours later.

### Western blot analysis

Cell lysates and pelleted virions or VLPs (recovered by centrifugation through 20% sucrose) were separated on NuPage Novex 4–12% Bis-Tris Mini Gels (Invitrogen). Proteins were blotted onto nitrocellulose membranes. Thereafter, the blots were probed with primary antibodies and a corresponding peroxidase conjugated secondary antibody and were developed with WestPico chemiluminescent detection reagents (Pierce). Alternatively, blots were probed with antibodies as above, followed by secondary antibodies conjugated to IRDye 800CW or IRDye 680. Fluorescent signals were detected and quantitated using Odyssey (LI-COR Biosciences).

### Microscopy

293T cells stably expressing DsRed-Clathrin light chain were seeded on 3.5-cm, glass-bottomed dishes coated with poly-L-Lysine (Mattek). The following day, they were transfected with a plasmid expressing AP180C, using Lipofectamine 2000. Cells were fixed 24 hrs later and observed by deconvolution microscopy using an Olympus IX70-based Deltavision microscopy suite as described previously [48].

To generate fluorescent VLPs for microscopic analysis, 293T cells stably expressing DsRed-clathrin LC were cotransfected with plasmids expressing codon-optimized HIV-1 Gag, or Gag-Pol along with a plasmid expressing codon optimized Gag-GFP at a ratio of 4∶1 or 8∶1. Forty-eight hours post transfection the culture supernatants were pelleted through a 20% sucrose cushion. The resulting VLPs were resuspended in PBS, 0.22 µm filtered, and diluted 1∶1 with PBS containing 3% paraformaldehyde. They were fixed overnight onto poly-L-Lysine coated glass bottom dishes and, after permeabilization with 1% Trition X-100 and 0.5% SDS, the VLPs were stained with anti-HIV capsid antibody, and analyzed via microscopy (Deltavision, Applied Precision). Alternatively, proviral plasmids pNL4-3 and pNL4-3 MA-YFP were transfected in a 1∶1 ratio into 293T expressing DsRed-clathrin LC, subjected to the above protocol but were visualized without immunostaining.

For scanning electronic microscopy studies, 293T cells were transfected with plasmids expressing codon optimized pCR3.1/SIVmac Gag-IRES-eGFP cassette and inspected by fluorescent and scanning electron microscopy using a Hitachi S4700 field emission SEM (University of Missouri Electron Microscopy Core Facility).

For transmission electron microscopy, samples were fixed using paraformaldehyde and glutaraldehyde, postfixed using osmium tetroxide and then dehydrated and embedded in Embed-812 (EMS). Following polymerization, approximately 65 nm sections were cut with an ultramicrotome and mounted on copper grids. Sections were stained with uranyl acetate followed by lead citrate and imaged using a FEI TECNAI G2 transmission electron microscope.

## Supporting Information

Figure S1 Quantitation of clathrin incorporation into virions from infected CEMx174 or MT4 cells and transfected 293T cells. T-cell lines were infected with HIV-1(NL4-3) at an MOI of ∼0.5, washed extensively, and progeny virions harvested 40 h later. Alternatively, 293T cells were transfected with the indicated amounts of the HIV-1(NL4-3) proviral plasmid and virions were harvested 40 h later. Cell and virion lysates were analyzed by Western blotting with anti-Gag and clathrin HC antibodies, and fluorescent detection reagents for quantitation of signals (LI-COR). Virions were concentrated 5-fold during harvest and this was taken into account when calculating the percentage of the total clathrin HC in the entire culture that was present in virions rather than cells, which is indicated for lanes 2, 4 and 7.
(TIF)

Figure S2 Effects of IN mutations on clathrin incorporation into HIV-1 virions. 293T cells were transfected with protease-active and various protease-defective (D25A) HIV-1 proviral plasmids, including those that were otherwise either wild-type, or bore point mutations in IN (E152K, N184L, F185K). Cells and virions were analyzed by Western blotting with anti-Gag and clathrin HC antibodies.
(TIF)

Figure S3 Depletion and sequestration of clathrin using siRNAs and AP180C. (A) Two examples of clathrin HC depletion using siRNA. 293T cells were cotransfected with an HIV-1 proviral plasmid and luciferase (siLuc) or clathrin HC (siClath. HC) targeting siRNAs as described in materials and methods. Cell lysates were probed with anti-clathrin HC and anti-tubulin antibodies. Western blot signals were quantitated using a LiCOR Odyssey scanner and the clathrin protein levels were reduced by 78±4%. (B) Clathrin sequestration using AP180C. 293T cells stably expressing DsRed-Clathrin LC (Red) cells were transfected with plasmids expressing FLAG-tagged AP180N (left panels) or AP180C (right panels) and subjected to immunofluorescent staining with an anti-FLAG antibody (green). Images were acquired using a deconvolution microscope and Optical sections at the center of the vertical dimension of the cell and at the cell-coverslip interface are displayed.
(TIF)

Figure S4 Effects of IN mutations on levels of Pol proteins (RT and IN) in HIV-1 virions. 293T cells were transfected with protease-active HIV-1 (NL4-3) proviral plasmids that were either wild-type, or bore point mutations (E152K, N184L, F185K) or a truncation (δCTD). Cells were analyzed by Western blotting with anti-Gag antibody, while virion lysates were probed with anti-Gag, anti-RT and anti-IN antibodies.
(TIF)

Figure S5 Effects of AP180C expression on incorporation of clathrin into HIV-1 virions. 293T cells were transfected with codon optimized HIV-1 GagPol expression plasmid and either an empty vector or increasing amounts of an AP180C expression plasmid. Cells and virion lysates were analyzed by Western blotting with anti-Gag, and anti-clathrin-HC antibodies.
(TIF)

Figure S6 Effects of AP180C expression on the Env protein in HIV-1 virions. 293T cells were transfected with HIV-1 (NL4-3) proviral plasmids and either an empty vector or plasmids expressing AP180-N or AP180-C, as indicated. Cells and virion lysates were analyzed by Western blotting with anti-Gag and anti-Env (gp120) antibodies.
(TIF)

Figure S7 Additional examples of scanning EM images of 293T cells transfected with plasmids expressing codon optimized WT or the indicated mutant SIVmac Gag-IRES eGFP cassettes.
(TIF)

Figure S8 Additional examples of scanning EM images of 293T cells transfected with plasmids expressing codon optimized SIVmac Gag-IRES eGFP cassettes in presence of AP180C.
(TIF)

Figure S9 DLL- mutations in MLV GagPol affect the viral protease in *cis* but not in *trans*. Cells were transfected with a fixed amount (50 ng) of plasmids expressing wild-type (left panels) or DLL-mutant (right panels) MLV GagPol and increasing amounts (0, 25, 50, 100, 200, 400, 800 ng) of wild-type or DLL-mutant MLV Gag-only expression plasmid. Cell lysates and virions were subjected to Western blot analysis and were probed with anti-p12 (upper 4 panels) and anti-capsid (lower 4 panels) monoclonal antibodies.
(TIF)

Figure S10 The MA-p12 junction can be cleaved by an MLV protease in *trans*. Cells were transfected with 6 µg of a plasmid expressing wild-type XMRV GagPol and 300 ng of a plasmid expressing wild-type or DLL-mutant MLV Gag. Cell lysates and virions were subjected to Western blot analysis and were probed with anti-p12 (upper panels) and anti-capsid (lower panels) monoclonal antibodies.
(TIF)

Figure S11 (A) Transmission electron microscopic analysis of cells transfected with a DLL-mutant MLV proviral plasmid, revealing particles of grossly normal morphology. (B) Western blot analysis of virions and VLPs used in the TRIM5 saturation assays (Figure 8I) to verify that approximately equivalent quantities of physical particles were used.
(TIF)

Table S1 Requirements for DsRed-clathrin LC incorporation into HIV-1 VLPs. GFP+ VLPs were generated by co-transfection of 293T/DsRed-clathrin-LC cells with Gag or GagPol expression plasmids and Gag-GFP in a 4∶1 ratio and filtered VLPs (0.22 µm) were applied to a microscope slide. Alternatively, a proviral plasmid encoding YFP embedded in the stalk region of MA was used (HIV-1 MA-YFP). See materials and methods for details. Three fields containing between 500 and 1500 GFP or YFP+ VLPs were evaluated for DsRed-clathrin LC incorporation using NIH IMAGE.
(DOC)

Table S2 Summary of the effects of mutations that block clathrin incorporation on retroviruses.
(DOC)
