# Supplementary figures and images for: Clathrin Facilitates the Morphogenesis of Retrovirus Particles
Source: PLoS Pathog. 2011 Jun 30;7(6):e1002119. doi: 10.1371/journal.ppat.1002119 (PMC3128127; doi:10.1371/journal.ppat.1002119)

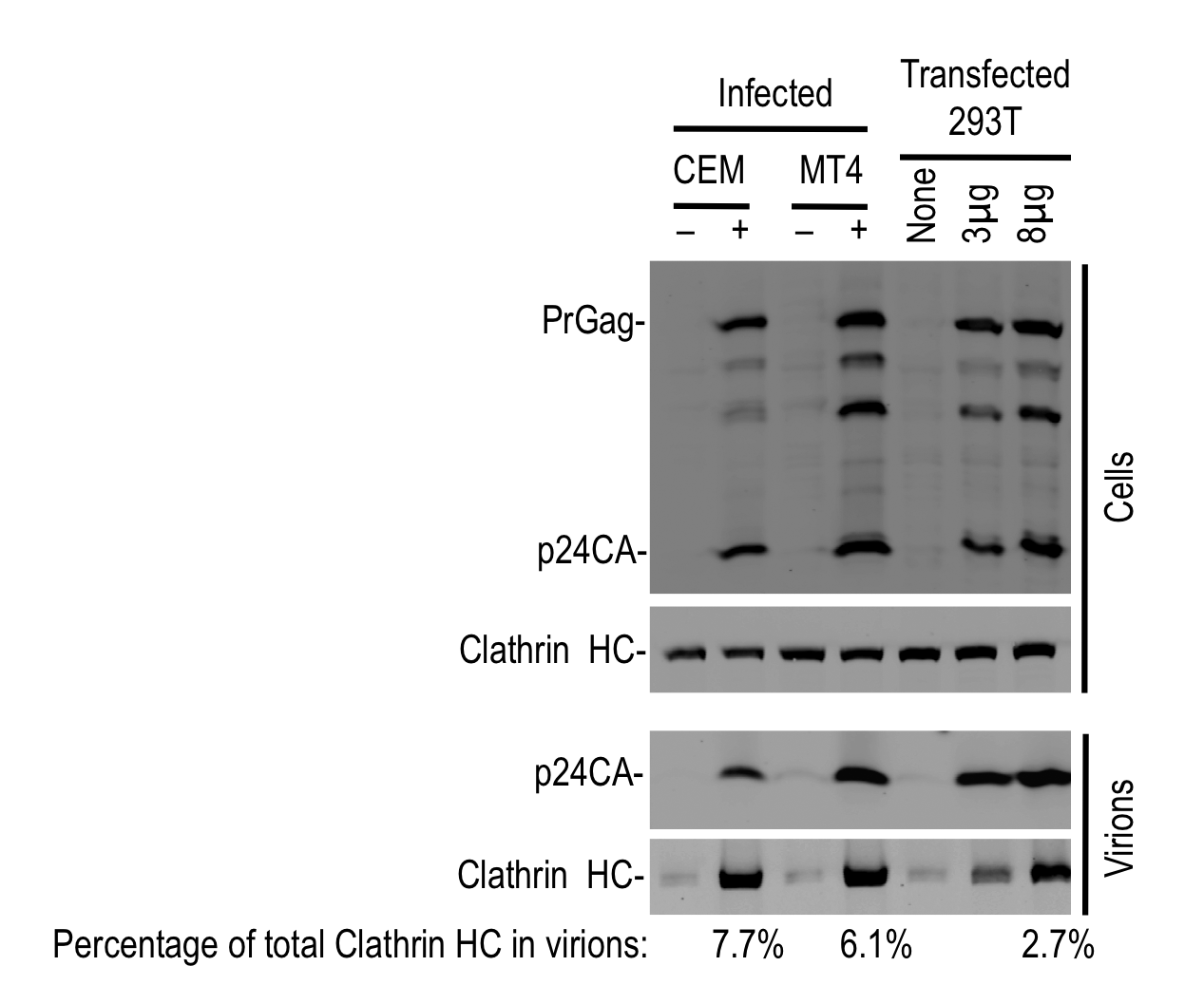

Supplement: Figure S1 — Quantitation of clathrin incorporation into virions from infected CEMx174 or MT4 cells and transfected 293T cells. T-cell lines were infected with HIV-1(NL4-3) at an MOI of ∼0.5, washed extensively, and progeny virions harvested 40 h later. Alternatively, 293T cells were transfected with the indicated amounts of the HIV-1(NL4-3) proviral plasmid and virions were harvested 40 h later. Cell and virion lysates were analyzed by Western blotting with anti-Gag and clathrin HC antibodies, and fluorescent detection reagents for quantitation of signals (LI-COR). Virions were concentrated 5-fold during harvest and this was taken into account when calculating the percentage of the total clathrin HC in the entire culture that was present in virions rather than cells, which is indicated for lanes 2, 4 and 7. (TIF) [file ppat.1002119.s001.tif]

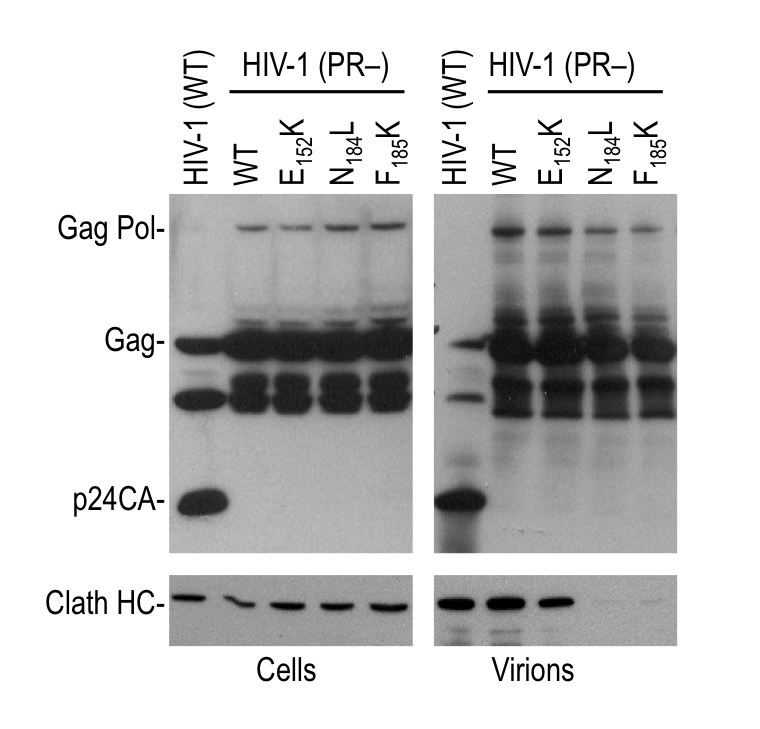

Supplement: Figure S2 — Effects of IN mutations on clathrin incorporation into HIV-1 virions. 293T cells were transfected with protease-active and various protease-defective (D25A) HIV-1 proviral plasmids, including those that were otherwise either wild-type, or bore point mutations in IN (E152K, N184L, F185K). Cells and virions were analyzed by Western blotting with anti-Gag and clathrin HC antibodies. (TIF) [file ppat.1002119.s002.tif]

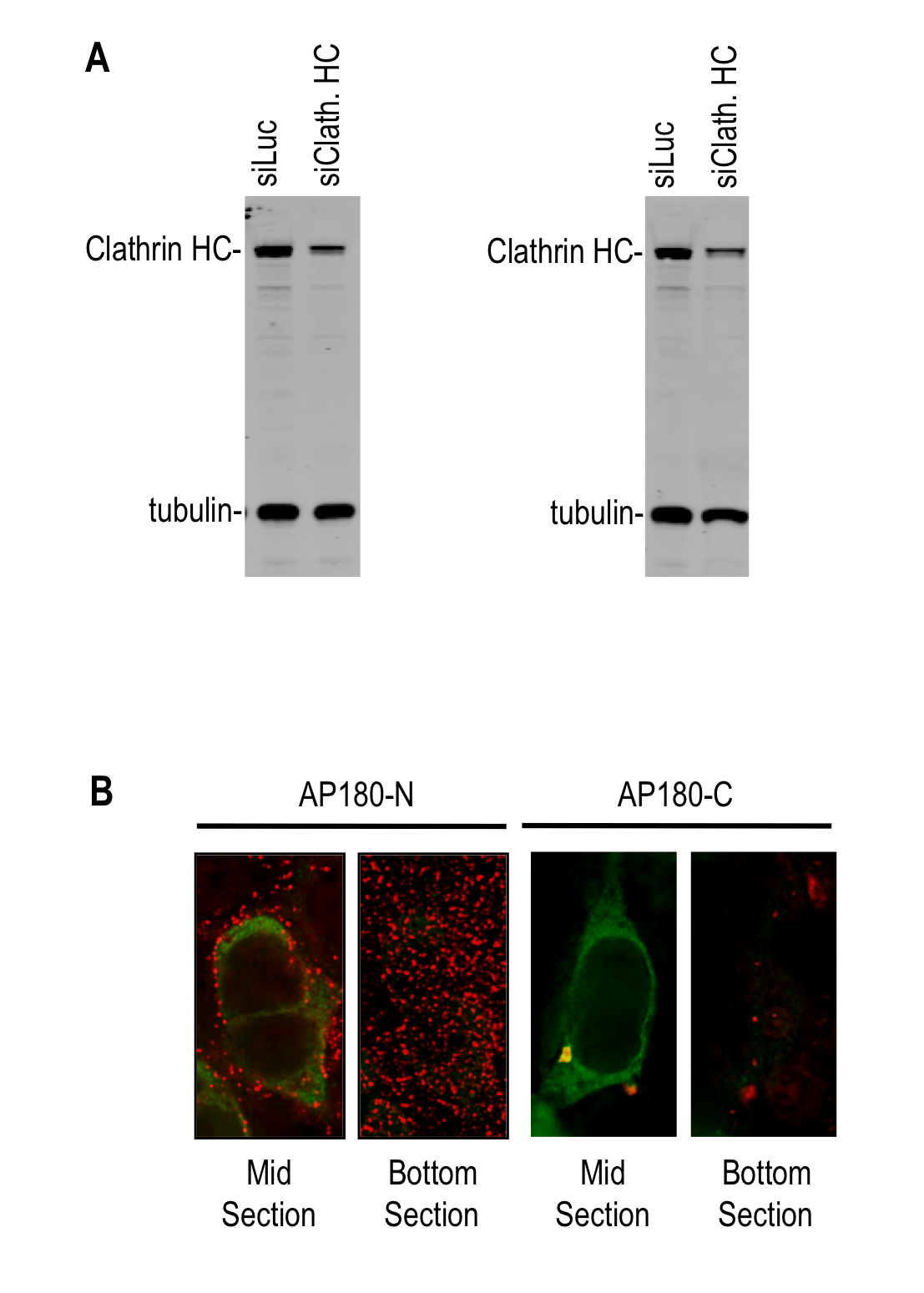

Supplement: Figure S3 — Depletion and sequestration of clathrin using siRNAs and AP180C. (A) Two examples of clathrin HC depletion using siRNA. 293T cells were cotransfected with an HIV-1 proviral plasmid and luciferase (siLuc) or clathrin HC (siClath. HC) targeting siRNAs as described in materials and methods. Cell lysates were probed with anti-clathrin HC and anti-tubulin antibodies. Western blot signals were quantitated using a LiCOR Odyssey scanner and the clathrin protein levels were reduced by 78±4%. (B) Clathrin sequestration using AP180C. 293T cells stably expressing DsRed-Clathrin LC (Red) cells were transfected with plasmids expressing FLAG-tagged AP180N (left panels) or AP180C (right panels) and subjected to immunofluorescent staining with an anti-FLAG antibody (green). Images were acquired using a deconvolution microscope and Optical sections at the center of the vertical dimension of the cell and at the cell-coverslip interface are displayed. (TIF) [file ppat.1002119.s003.tif]

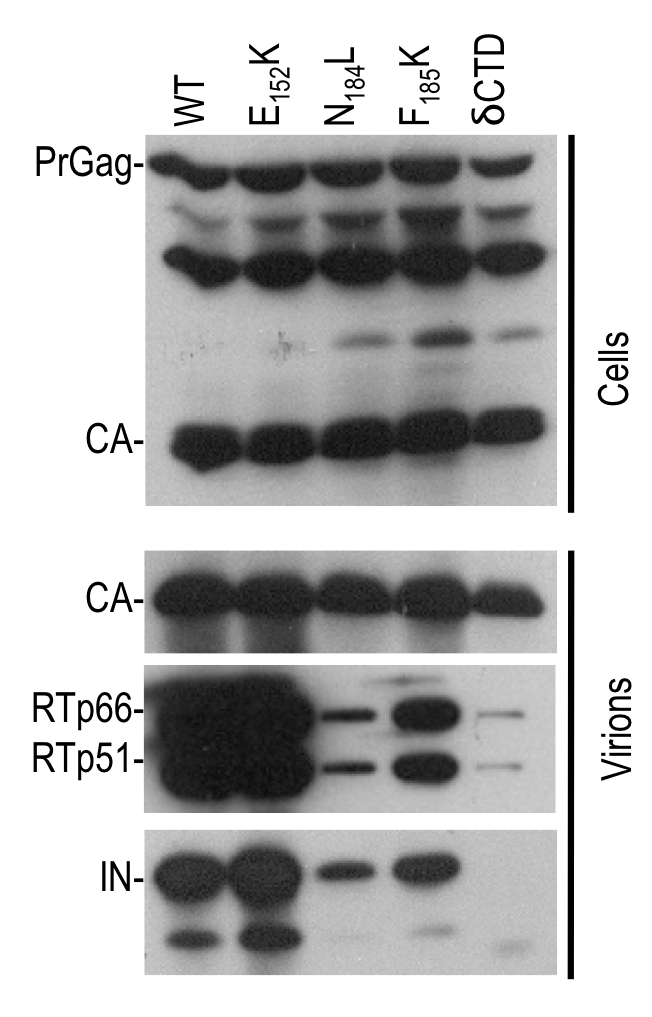

Supplement: Figure S4 — Effects of IN mutations on levels of Pol proteins (RT and IN) in HIV-1 virions. 293T cells were transfected with protease-active HIV-1 (NL4-3) proviral plasmids that were either wild-type, or bore point mutations (E152K, N184L, F185K) or a truncation (δCTD). Cells were analyzed by Western blotting with anti-Gag antibody, while virion lysates were probed with anti-Gag, anti-RT and anti-IN antibodies. (TIF) [file ppat.1002119.s004.tif]

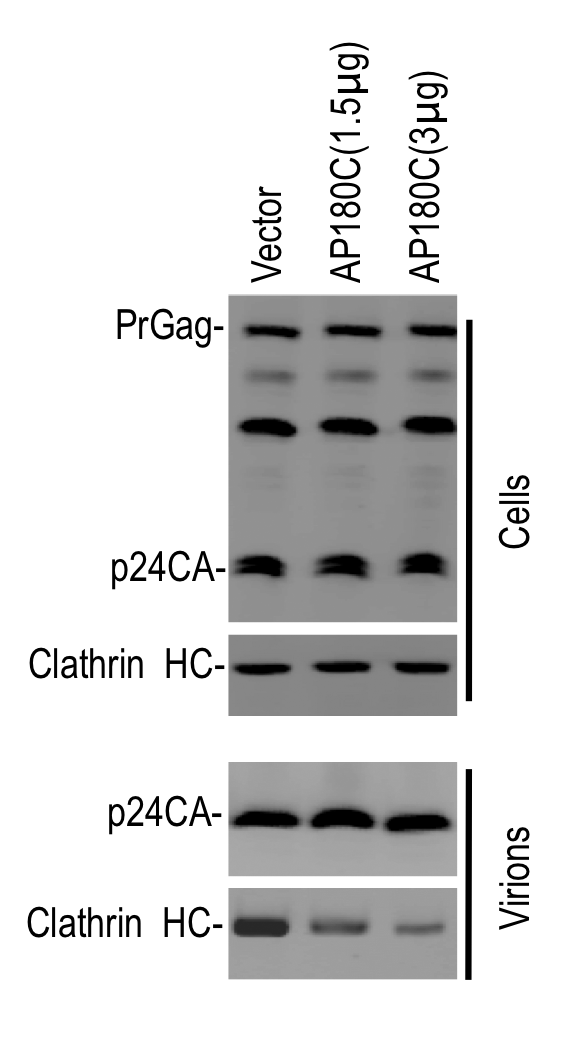

Supplement: Figure S5 — Effects of AP180C expression on incorporation of clathrin into HIV-1 virions. 293T cells were transfected with codon optimized HIV-1 GagPol expression plasmid and either an empty vector or increasing amounts of an AP180C expression plasmid. Cells and virion lysates were analyzed by Western blotting with anti-Gag, and anti-clathrin-HC antibodies. (TIF) [file ppat.1002119.s005.tif]

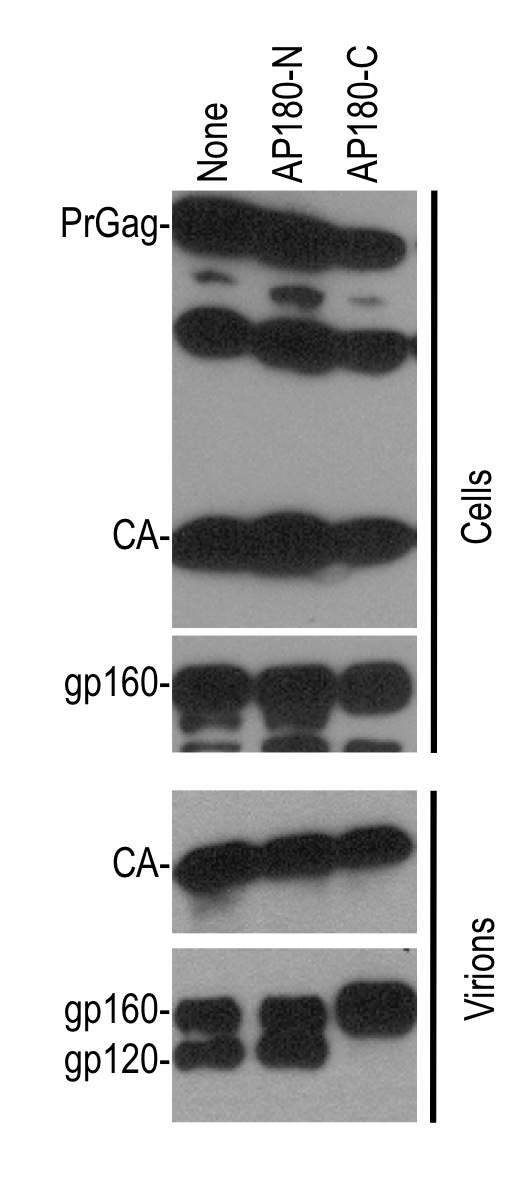

Supplement: Figure S6 — Effects of AP180C expression on the Env protein in HIV-1 virions. 293T cells were transfected with HIV-1 (NL4-3) proviral plasmids and either an empty vector or plasmids expressing AP180-N or AP180-C, as indicated. Cells and virion lysates were analyzed by Western blotting with anti-Gag and anti-Env (gp120) antibodies. (TIF) [file ppat.1002119.s006.tif]

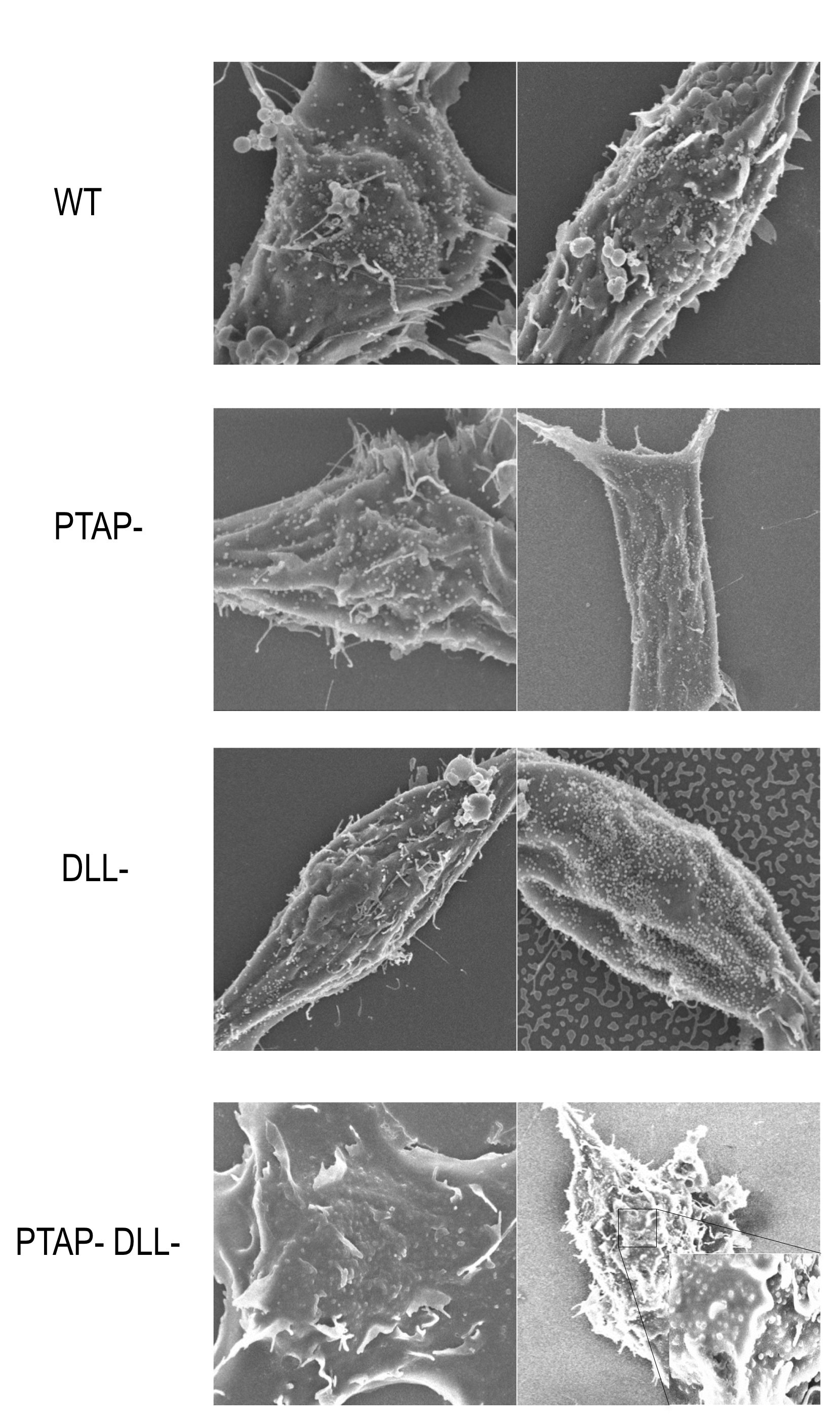

Supplement: Figure S7 — Additional examples of scanning EM images of 293T cells transfected with plasmids expressing codon optimized WT or the indicated mutant SIVmac Gag-IRES eGFP cassettes. (TIF) [file ppat.1002119.s007.tif]

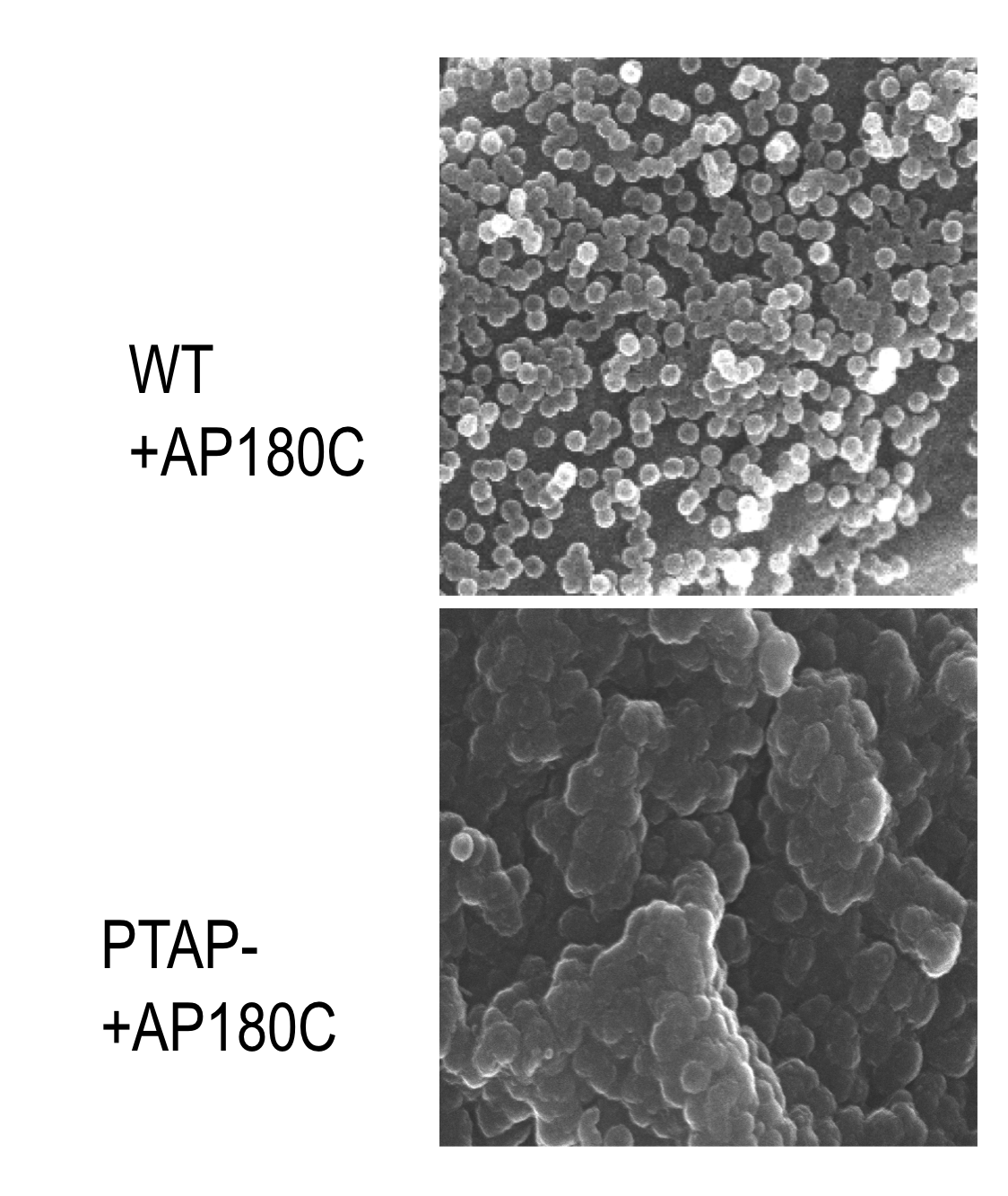

Supplement: Figure S8 — Additional examples of scanning EM images of 293T cells transfected with plasmids expressing codon optimized SIVmac Gag-IRES eGFP cassettes in presence of AP180C. (TIF) [file ppat.1002119.s008.tif]

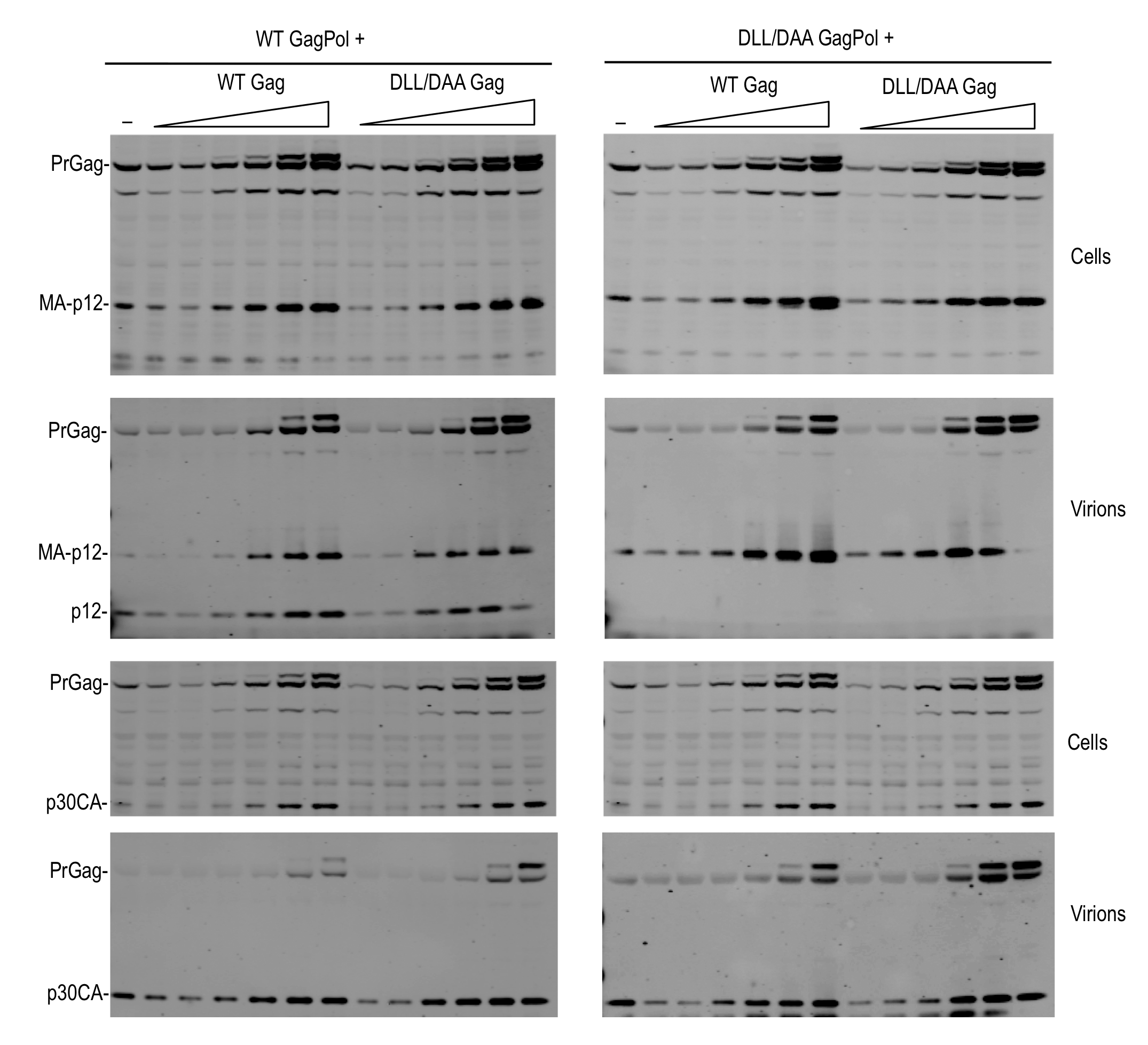

Supplement: Figure S9 — DLL- mutations in MLV GagPol affect the viral protease in cis but not in trans. Cells were transfected with a fixed amount (50 ng) of plasmids expressing wild-type (left panels) or DLL-mutant (right panels) MLV GagPol and increasing amounts (0, 25, 50, 100, 200, 400, 800 ng) of wild-type or DLL-mutant MLV Gag-only expression plasmid. Cell lysates and virions were subjected to Western blot analysis and were probed with anti-p12 (upper 4 panels) and anti-capsid (lower 4 panels) monoclonal antibodies. (TIF) [file ppat.1002119.s009.tif]

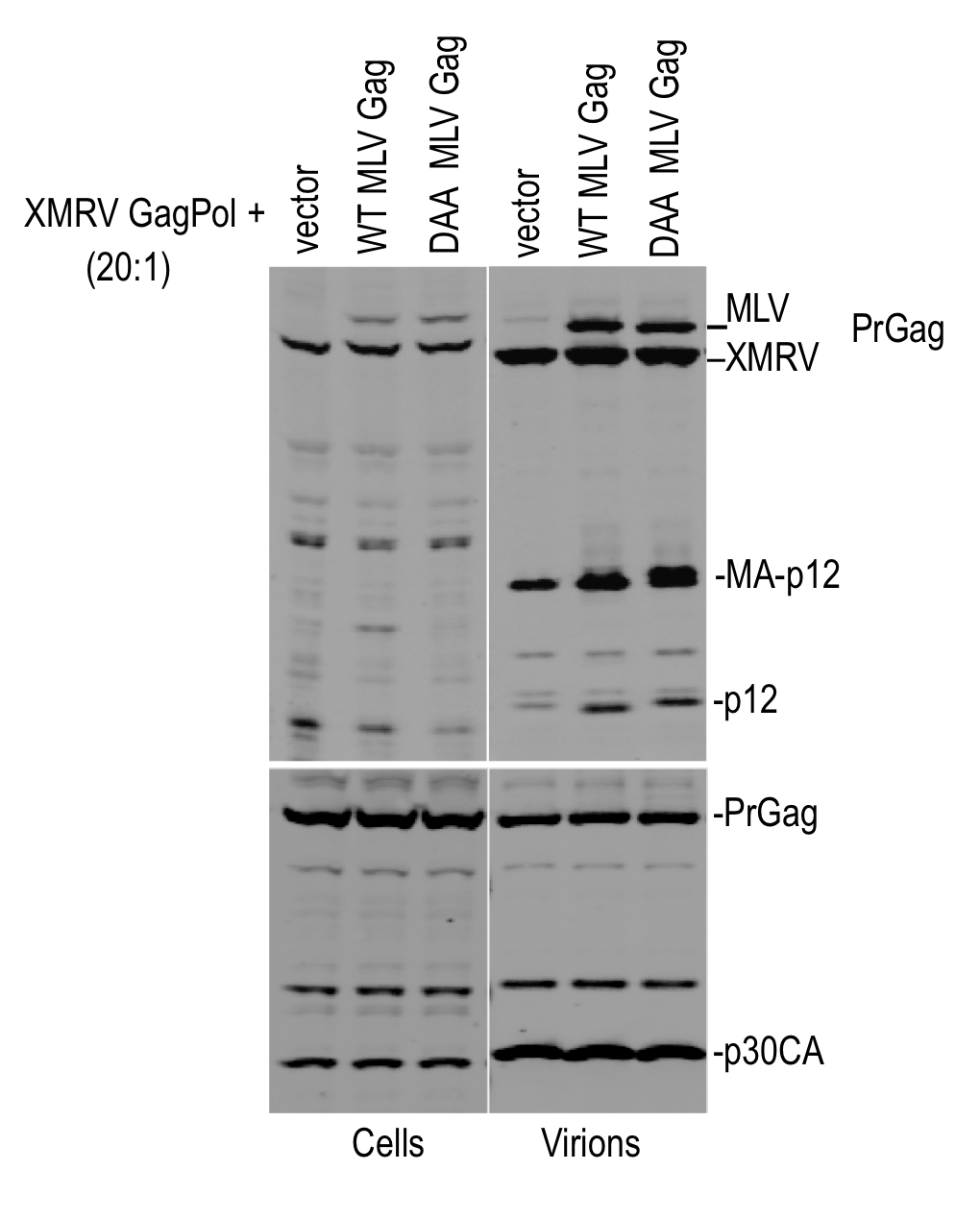

Supplement: Figure S10 — The MA-p12 junction can be cleaved by an MLV protease in trans. Cells were transfected with 6 µg of a plasmid expressing wild-type XMRV GagPol and 300 ng of a plasmid expressing wild-type or DLL-mutant MLV Gag. Cell lysates and virions were subjected to Western blot analysis and were probed with anti-p12 (upper panels) and anti-capsid (lower panels) monoclonal antibodies. (TIF) [file ppat.1002119.s010.tif]

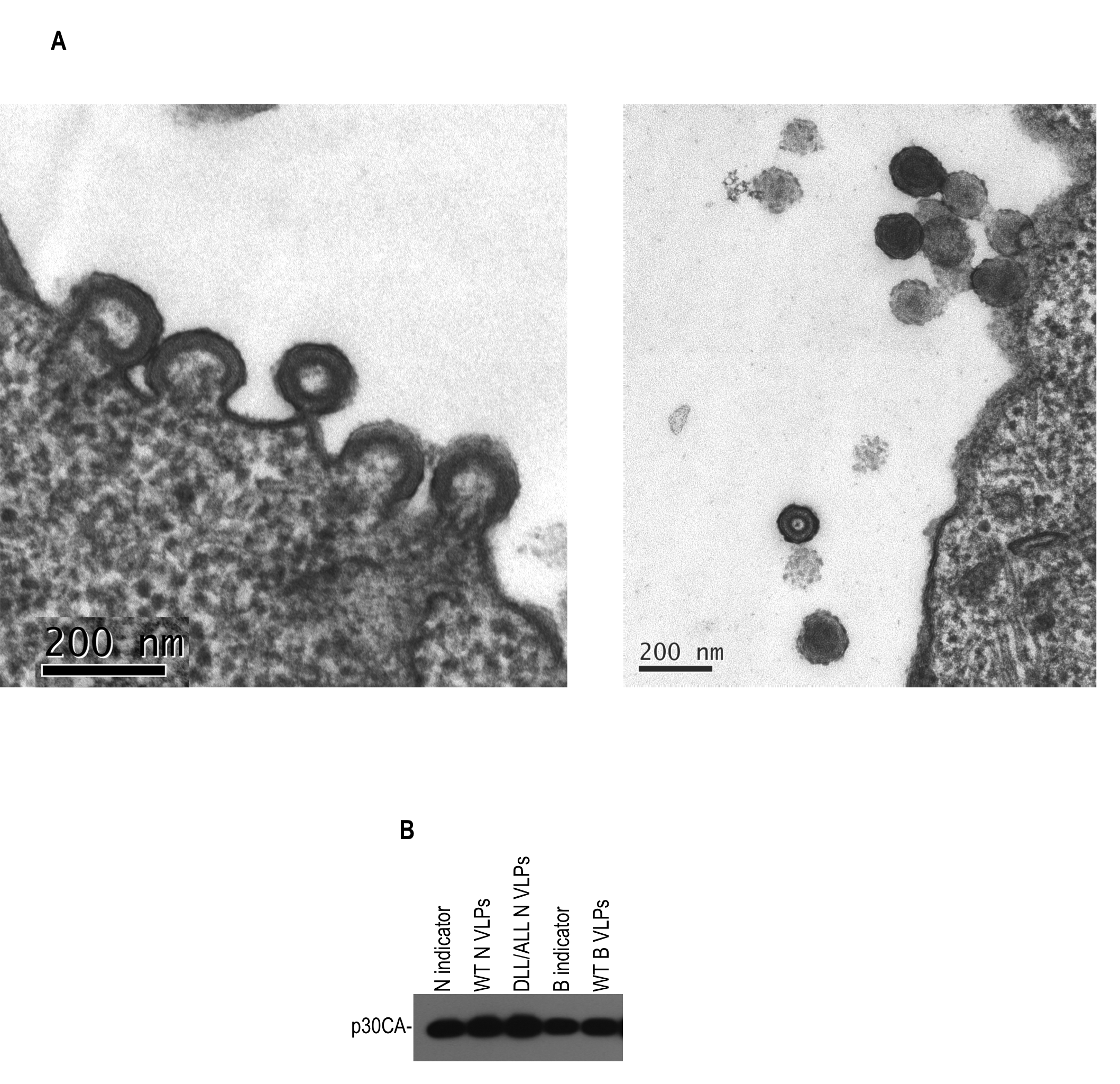

Supplement: Figure S11 — (A) Transmission electron microscopic analysis of cells transfected with a DLL-mutant MLV proviral plasmid, revealing particles of grossly normal morphology. (B) Western blot analysis of virions and VLPs used in the TRIM5 saturation assays (Figure 8I) to verify that approximately equivalent quantities of physical particles were used. (TIF) [file ppat.1002119.s011.tif]
