# Supplementary material for: Clathrin Facilitates the Morphogenesis of Retrovirus Particles
Source: PLoS Pathog. 2011 Jun 30;7(6):e1002119. doi: 10.1371/journal.ppat.1002119 (PMC3128127; doi:10.1371/journal.ppat.1002119)
Supplement: Table S1 — Requirements for DsRed-clathrin LC incorporation into HIV-1 VLPs. GFP+ VLPs were generated by co-transfection of 293T/DsRed-clathrin-LC cells with Gag or GagPol expression plasmids and Gag-GFP in a 4∶1 ratio and filtered VLPs (0.22 µm) were applied to a microscope slide. Alternatively, a proviral plasmid encoding YFP embedded in the stalk region of MA was used (HIV-1 MA-YFP). See materials and methods for details. Three fields containing between 500 and 1500 GFP or YFP+ VLPs were evaluated for DsRed-clathrin LC incorporation using NIH IMAGE. (DOC) [file ppat.1002119.s012.doc]

Table S1. Requirements for DsRed-clathrin LC incorporation into HIV-1 VLPs

| Viral Proteins | % DsRed+ VLPs |
| --- | --- |
| Gag | 0.4±0.2 |
| GagPol | 32±1 |
| GagPol (PR-) | 37±1 |
| GagPol (δIN CTD) | 1.3±0.2 |
| GagPol (δRT) | 0.2±0.3 |
| GagPol (+Efavirenz) | 1.5±1.9 |
| GagPol (PR-, +Efavirenz) | 6±1.9 |
| HIV-1 (MA-YFP) | 76±17 |
