# Supplementary material for: Clathrin Facilitates the Morphogenesis of Retrovirus Particles
Source: PLoS Pathog. 2011 Jun 30;7(6):e1002119. doi: 10.1371/journal.ppat.1002119 (PMC3128127; doi:10.1371/journal.ppat.1002119)
Supplement: Table S2 — Summary of the effects of mutations that block clathrin incorporation on retroviruses. (DOC) [file ppat.1002119.s013.doc]

**Table S2. Summary of the effects of mutations that block clathrin incorportation on retroviruses**

| Retrovirus | Protein or motif responsible for clathrin recruitment | Effects of mutations that block clathrin incorporation |
| --- | --- | --- |
| HIV-1 | Pol (RT and IN) | Reduced virion infectivity  Reduced Pol (RT and IN) levels in cells and virions (only when protease active) |
| SIVmac | Gag (p6) DLL motifs  Pol (minor contribution to overall clathrin incorporation) | Minor effects on virion infectivity in single cycle replication assays, dramatic effects in spreading replication assays  Defect in generation and release of spherical particles (when combined with L-domain mutation)  Reduced Gag levels when combined with L-domain mutations (only when protease active)  Effects of Gag DLL mutations suppressed by ALIX overexpression  Reduced virion infectivity  Reduced Pol levels in cells |
| MuLV | Gag (p12) DLL motif | Reduced virion infectivity  Reduced p12 protein in virions  Aberrant capsid morphology or reduced capsid stability (impaired TRIM5 saturation) |
| MPMV | Gag (pp24) DLISLD motif | Reduced virion infectivity |
